# Development of a human-analogue, 3-symptom domain Dog ADHD and Functionality Rating Scale (DAFRS)

**DOI:** 10.1038/s41598-024-51924-9

**Published:** 2024-01-20

**Authors:** Barbara Csibra, Nóra Bunford, Márta Gácsi

**Affiliations:** 1 Department of Ethology, Institute of Biology, Eötvös Loránd University, Pázmány Péter sétány 1/C, Budapest, 1117 Hungary; 2 Doctoral School of Biology, Institute of Biology, ELTE Eötvös Loránd University, Pázmány Péter sétány 1/C, Budapest, 1117 Hungary; 3 Clinical and Developmental Neuropsychology Research Group, Research Centre for Natural Sciences, Institute of Cognitive Neuroscience and Psychology, Magyar tudósok Körútja 2, Budapest, 1117 Hungary; 4 ELKH-ELTE Comparative Ethology Research Group, Pázmány Péter sétány 1/C, Budapest, 1117 Hungary

**Keywords:** Animal behaviour, ADHD

## Abstract

The family dog, in its natural environment, exhibits neuropsychological deficits redolent of human psychiatric disorders, including behaviours that are similar to human attention-deficit/hyperactivity disorder (ADHD) symptoms. Based on standard questionnaire methods in humans, we aimed to develop and validate a detailed, psychometrically improved tool to assess owner views on relevant dog behaviours. We modified available questionnaires by adding items that allow for separate analysis of impulsivity, and items on functional impairment. We collected data from 1168 owners for different validation steps of the new questionnaire and, similarly to assessment of humans where teachers also evaluate as an expert control, we collected data from dog trainers. Exploratory and confirmatory factor analysis revealed 3 factors: inattention (IA), hyperactivity (H) and impulsivity (I), corresponding to all three human symptom dimensions in dogs. Test–retest analyses showed excellent agreement between measurements for all factors. Similarly to findings with humans, trainer-owner rating comparisons showed fair (IA) to moderate (H, I) agreement. As in humans, greater ADHD scores were associated with greater functional impairment scores. We suggest that in dogs, similarly to humans, parallel examination of (extreme) ADHD and functional impairment scores could help distinguish diagnosable individuals, after further validation of the questionnaire using a relevant behaviour test.

## Introduction

Attention-deficit/hyperactivity disorder (ADHD) is an often lifelong and prevalent neurodevelopmental disorder in humans^1–3^, characterized by developmentally inappropriate inattention, hyperactivity and impulsivity symptoms^4^ as well as functional impairment in the academic, occupational, and social domains^5,6^. Inattention is defined by a relatively low ability to sustain concentration in tasks, distractibility, forgetfulness, disorganization, avoiding tasks that require sustained effort^4,7^. Hyperactivity is characterized by an excessive increase in motor activity, often manifesting as persistent restlessness, fidgeting, and difficulty in maintaining stillness in situations when remaining still or seated is expected^4,8^. Impulsivity is characterized by a tendency to act on immediate urges or stimuli without sufficient forethought and conscious judgement compared to individuals with similar levels of knowledge and ability^4,9^. It encompasses behaviours marked by insufficient sampling of sensory evidence (reflection impulsivity), a failure of motor inhibition (impulsive action), intolerance of delay to rewards (impulsive choice) in the context of decision-making^10^.

In humans, a multi-informant and -method assessment battery is typically employed for assessment of ADHD, involving relevant informants (e.g., the child’s parent and/or teacher) as well as clinical interviews, behavioural observation, behaviour rating scales, and clinic-based testing^11^. As children less able to provide reliable and valid information about their own behaviour and functioning^12^, especially in case of externalizing symptoms, information about the behaviour of the child in different settings is obtained from parents and teachers^11,13^.

Although various questionnaires are available to assess ADHD symptoms and related impairment in humans, only few have been updated to align with DSM-5^14^ criteria. The ADHD Rating Scale-5^15^ is a 30-item measure of parent (and teacher) ratings of child DSM-5 ADHD symptoms over the past 6 months, consisting of two symptom subscales: inattention (9 items) and hyperactivity-impulsivity (9 items) as well as two impairment subscales for inattention items (6 items) and hyperactivity/impulsivity items (6 items) causing difficulties in everyday functioning in: relationships with family members and teachers, peer relationships, academic functioning, behavioural functioning, homework functioning, and self-esteem^15^.

Although, hyperactivity-impulsivity is characteristically assessed as one dimension of ADHD, evidence indicates that the factor structure of ADHD symptoms may change during development, including hyperactivity and impulsivity symptoms diverge at some point in adolescence^16^. Data show that impulsivity attenuates at a slower rate than hyperactivity during the transition to adolescence^17^, and this may explain why during adulthood a 3-factor structure of ADHD (inattention, hyperactivity, and impulsivity) is a better fit than the 2-factor structure (inattention and hyperactivity/impulsivity) applied for children^17–21^.

Animal models can provide insight into aspects of neurodevelopmental and mental disorders that cannot be ethically assessed or practically manipulated in humans^22^. A large body of work indicates the domestic dog is a promising animal model of neurological disorders including autism^23^, Alzheimer's disease^24^ and ADHD-like behaviour^25–33^. Contrary to rodents, dogs naturally exhibit phenotypic variability in ADHD symptoms^32,33^ and show similar gene-phenotype associations^34–38^ as well as functional impairments^39,40^ as humans, consistent with criteria for being a valid model for disorders in humans^41^.

Apart from using the dog as an animal model of human ADHD, in veterinarian practice, there are more and more cases reporting that dogs display ADHD-like behaviours and symptoms, resembling human ADHD^25,42,43^. Although there is no clear consensus on the definition of ADHD-like behaviour or whether ADHD exists in dogs ^42,43^, veterinarians diagnose and medicate dogs with Hypersensitivity-Hyperactivity syndrome (HSHA)^42^, where the proposed definition of HSHA covers three main symptoms: hyperactivity, lack of satiety, and shorter sleep duration with normal cycles^42^. Although HSHA is often reported by veterinarians as an “ADHD-like syndrome” in dogs or “canine ADHD”^42^, it remains uncertain whether HSHA is a distinct or related condition compared to “ADHD” in dogs (if ADHD exists). The published methodology lacks differential diagnosis and the assessment of to what degree it parallels the clinical manifestations of human ADHD.

Several questionnaires have been developed to assess ADHD-related characteristics in dogs (for a review see^28^), with an extended and revised version^28^ of the Dog ADHD Rating Scale—Dog ARS^32^ exhibiting evidence of acceptable psychometric properties^28^, including stable factor structure (inattention and hyperactivity-impulsivity) and temporal stability^28^. In its present form however, the Dog ARS, is not appropriate to detect diagnosable individuals with ADHD. On the one hand, it does not include functionality assessment, which would be essential to develop diagnostic criteria for dogs. On the other hand, the Dog ARS has been exclusively applied to family dogs as a general questionnaire to assess inattention, activity, and impulsivity, it was not intended to be used directly to diagnose dogs (i.e., presumably in all successful applications of the questionnaire, typical dogs were measured, or at least samples specifically selected/grouped for the extremities in ADHD-like behaviour were not utilised).

Further, there are limitations with regard to comparability across human ADHD rating scales and the Dog ARS: in humans, hyperactivity and impulsivity are measured separately^21,44–49^. In the absence of empirical data, it is unknown whether these are separable in dogs and, as such, the hyperactivity-impulsivity distinction is not reflected in the Dog ARS. Exploration of the differentiability of hyperactivity and impulsivity would require targeted items.

Moreover, human diagnostic guidelines suggest that behaviour should be assessed in multiple contexts as part of the evaluation process, thereby involving data from multiple informants, such as parents and teachers^50–52^. Both for purposes of comparability across human and dog research and to obtain a comprehensive view of the dog’s behaviour, it appears warranted to involve experts (e.g., dog trainers) in the evaluation process in canine ADHD research.

Finally, a central element of human ADHD diagnosis is the presence of functional impairments, i.e., that the symptoms interfere with, or reduce the quality of, academic, social, or occupational functioning (DSM-5^14^). Although there is evidence that comparable to children, dogs show functional impairments^39,40^, measuring ADHD-related functioning specifically has not been given a prominent role in canine research. Beyond assessing frequency of symptoms, measuring functional impairment is key both to better understand the consequences of behaviour problems and to determine whether dogs can be diagnosed with ADHD which, in turn, is important for the proper treatment and well-being of affected animals. Behavioural problems linked to ADHD traits can negatively impact the human-animal relationship^53,54^ and result in the animal being relinquished to an animal shelter or even euthanised. Accordingly, a shift towards methods that better assist diagnosis and are more precise is essential to improve both owners’ and dogs’ quality of life.

In the current study, based on assessment methods used with humans, our aim was to develop and validate a detailed and improved questionnaire of owner-reported, ADHD-relevant dog behaviours with questions that allow for separate assessment of hyperactivity and impulsivity as well as functional impairment. For the first time, we also included experts (dog trainers) in the evaluation process. Further, our aims were to evaluate various indices of the psychometric properties of the DAFRS (Dog ADHD and Functionality Rating Scale)—i.e., ambiguous items, factor structure, internal consistency, interrater and test–retest reliability, as well as convergent validity across multiple samples of domestic dogs.

We organised our objectives into six specific aims and corresponding sub-aims, summarised in Table 1.

**Table 1:** Summary of the aims, corresponding methods and samples used in the present study.

Aim/Question	Method	Sample
Aim 1. Compile a new questionnaire; DAFRS a. Clarification of ambiguous items in the Dog ARS b. Formulate additional items that allow for separate assessment of impulsivity c. Add items that allow for assessment of functional impairment	a. Reword the Dog ARS questionnaire items with > 5% IDK response rate b. & c. Include new questions based on human/dog questionnaires, literature and experts’ view	a. Csibra et al.^28^ data (Dog ARS IDK-O (*N* = 520) and Dog ARS IDK-T (*n* = 86)) b. & c. DAFRS sample (*N* = 1168)
Aim 2. Identify and eliminate ambiguous items/functionality items in the DAFRS	‘IDK’ option on an independent sample, drop DAFRS items with > 5% IDK response rate	DAFRS + IDK-O (*N* = 210)
Aim 3. Examine the factor structure—including to determine whether hyperactivity can be distinguished from impulsivity in dogs—and the internal consistency of the DAFRS	Exploratory factor analysis (half sample) and Confirmatory factor analysis (other half), Cronbach’s alpha on CFA data, report response distributions	DAFRS sample (*N* = 1168) EFA: *n* = 584 (random half of *N* = 1168) CFA: *n* = 584 (other half) Cronbach’s alpha and CFA on factors, report response distributions (*N* = 1168)
Aim 4. Examine the test–retest reliability of the DAFRS	Intraclass correlation coefficient	DAFRS subsample (*n* = 231/1168)
Aim 5. Collect data from owners and trainers on the DAFRS	Intraclass correlation coefficient, report response distributions	DAFRS + IDK: Owner (IDK-O) vs. Trainer (IDK-T) (*N* = 70)
Aim 6. Examine evidence of the convergent validity of the DAFRS: Differences across age, sexes, and associations with functional impairment	Correlations between sex, neutering status, age, factor scores and relevant functional impairment scores	DAFRS (*N* = 1168)

A: aim, Q: question, Dog ARS: previously obtained data by Vas et al.^32^, Csibra et al.^28^ data: previously obtained data by Csibra et al.^28^, IDK: “I don’t know”, Dog ARS IDK-O: Csibra et al. dataset on the Dog ARS with “I don’t know” option for owners, Dog ARS IDK-T: Csibra et al. dataset current dataset on the Dog ARS with “I don’t know” option for trainers, DAFRS: current dataset on Dog ADHD and Functionality Rating Scale, ICC: Intraclass correlation coefficient; EFA: Exploratory factor analysis, CFA: Confirmatory factor analysis, DAFRS IDK-O: current dataset on ADHD and Functionality Rating Scale with I don’t know option for owners, DAFRS IDK-T: current dataset on ADHD and Functionality Rating Scale with I don’t know option for owners for trainers.

## Results

### Aim 1—Formulating a new questionnaire: the Dog ADHD and Functionality Rating Scale (DAFRS)

The questionnaire has face validity as it was constructed in collaboration with human clinical experts and researchers with vast experience in ADHD and associated problems, and with dog behaviour experts such as veterinarians specializing in dog behaviour (including a diplomat from ECAWBM and a European Veterinary Specialist in Behavioral Medicine) as well as ethologists and dog trainers. The domains were chosen first (inattention, hyperactivity and impulsivity) and items reflecting these were then selected until it was judged that each subdomain was sufficiently covered. The initial questionnaire had 15 items on inattention, 14 items on hyperactivity, 13 items on impulsivity. Initially, we formed 28 items in total on functional impairments. For the originally formed ADHD and functionality items see supplementary material, appendix C.

### Aim 2—Identify and eliminate ambiguous items/functionality items in the DAFRS

To filter out ambiguous items in the DAFRS questionnaire, we used the proportion of IDK responses to determine which items need to be dropped out before the exploratory factor analyses. Data on IDK answer proportions were ≤ 3.8% except for six questions: item 9, item 18, item 22, item 31, item 34, item 39, where the reported proportions were above 5%. These items were eliminated before the exploratory factor analyses to exclude ambiguous questions, which could have led to misleading results (Aim 3). In case of functionality items, the propo±rtion of IDK responses by owners were ≤ 2.9% on all items.

### Aim 3—Examine the factor structure and the internal consistency of the DAFRS

As this is the first psychometric analysis on the developed questionnaire, we began by conducting an exploratory factor analysis (EFA) in order to determine if the items were measuring one large construct (e.g., ADHD-like behaviours) or were measuring several independent yet related constructs (e.g., inattention, hyperactivity and impulsivity). The EFA was conducted with the 36 Likert-type items out of 42 items regarding inattention, hyperactivity and impulsivity, as in the previous part items which represented with high proportion IDK response rate (> 5%) dropped out from the questionnaire before the EFA (Aim 2). Half of the total sample, *n* = 584 randomly selected participants were used for EFA. As confirmatory factor analysis (CFA) requires a parallel sample to EFA, the other half of the sample (*n* = 584) was used in the CFA. Using oblique rotation, results showed that maintaining four factors produced the most adequate fit while maintaining the highest discrimination between item loadings and obtaining the greatest conceptual clarity between the factors. Based on the item loadings, the factors appeared to involve Inattention (e.g., has difficulties with learning, because he/she does not pay attention), Hyperactivity (e.g., fidgets, bustles), Impulsivity (e.g., has no self-control) and interestingly Vocalisation (e.g., cannot be quiet, whines or barks a lot even when there is nothing special to evoke this). However, several items loaded onto both factors with a similar magnitude. This can cause difficulty in terms of fitting a model using CFA as well as create problems with scoring and interpretation. Therefore, we began the process of item reduction, where items are eliminated with low loading (< 0.5 on any subscale) or sat on multiple components with comparable absolute loadings (> 0.40 on two or more subscales). After each item was deleted, the EFA was rerun to estimate new factor loadings. Using this process, 16 items were eliminated from the measure (Items 2, 5, 6, 10, 11, 15, 16, 19, 21, 28, 32, 35, 36, 37, 40 and 41). After the systematic elimination of items, four factors emerged: Inattention, Hyperactivity, Impulsivity and Vocalisation. As Vocalisation factor contained only 2 items (Item 8: Cannot be quiet, whines or barks a lot even when there is nothing special to evoke this; and Item 30: If your dog starts to bark or whine, it is difficult to silence him/her) and evidence-based guidelines for scale development suggest a minimum of three items per factor to consider a factor interpretable^55^, thus we did not consider Vocalisation as a factor, and eliminated the two items from the EFA and CFA measure but later, we included the total score of these two items in the functionality measures, as a “vocalisation” variable (see Aim 6.). All item loadings were greater than 0.5 at the end of the elimination procedure. The final resulting EFA produced three factors: Inattention (Items 12, 24, 25, 26, 27 and 29), Hyperactivity (Items 1, 14, 20, 23 and 33) and Impulsivity (Items 3, 4, 7, 13, 17, 38, and 42), see Table 2 for item descriptions. The three factors (factor 1 [Impulsivity], factor 2 [Inattention]) and factor 3 [Hyperactivity] see Table 2) accounting for 53.1% of the total variance (characterised by eigenvalues > 1). The first factor (Impulsivity), explains 20.1%, the second (Inattention) 18.9% and the third factor (Hyperactivity) 14.1% of the total variance.

**Table 2:** DAFRS items and factor loadings following exploratory factor analysis.

Items (DAFRS)	Factor loadings		
	IA	I	H
24. Has difficulties with learning, because he/she does not pay attention	**0.856**	0.021	0.047
26. Performs poorly on tasks that require a lot of thinking	**0.836**	− 0.011	0.038
25. When your dog is asked to perform a task, he/she is reluctant to comply or withdraws from the situation	**0.816**	− 0.021	− 0.042
27. Your dog has difficulties solving tasks (i.e., makes many mistakes) that it is familiar with and has practised a lot before	**0.671**	0.072	0.051
29. Quickly loses interest	**0.562**	0.178	− 0.145
12. Has difficulties concentrating	**0.553**	0.343	− 0.074
7. Has no self-control	0.035	**0.782**	0.052
38. Is difficult to control and handle	0.166	**0.757**	− 0.027
4. Once your dog "gets going", it is difficult to hold him/her back or stop	− 0.079	**0.728**	0.137
17. Is difficult to calm down	0.101	**0.705**	0.029
3. Becomes very excited when facing a new, mildly stressful situation (e.g., facing a new situation/place or meeting new people/dogs)	− 0.145	**0.633**	0.109
42. Reacts rashly to new stimuli, without considering the consequences	0.193	**0.623**	− 0.007
13. Is excessive, unrestrained, rampant	0.097	**0.523**	0.330
33. Would always play and run	− 0.053	0.079	**0.760**
14. Is rarely calm, even in familiar places, calm situations (e.g., at home)	0.127	0.108	**0.723**
20. Active even after fatiguing or hard exercise/work	− 0.065	0.041	**0.711**
1. Fidgets, bustles	− 0.149	0.261	**0.641**
23. Sleeps little, is active even during the night*	0.276	− 0.329	**0.585**

* = Item 23 was excluded after confirmatory factor analyses. In addition to inattention, hyperactivity, and impulsivity, a fourth factor, “Vocalisation” emerged in our analysis. Even though we did not retain it as a separate factor—as it contained only two items—, we recommend including these as functionality items, as excessive vocalisation is likely related to ADHD. The two vocalisation items had loadings of− 0.072 (IA), − 0.026 (I), 0.068 (H), 0.814 (V) for Item 8 and 0.033 (IA), − 0.014 (I), − 0.075 (H), 0.819 (V) for Item 30, respectively. Boldface font indicates the final factor solution following exploratory factor analyses.

DAFRS Dog ADHD and Functionality Rating Scale, Owner-report form. Subscales: *IA* inattention, *H* hyperactivity, *I* Impulsivity.

To assess the fit of the model produced by the EFA with another sample, we used CFA with the remaining 584 participants’ data. Final model fit was approaching excellent levels across fit indices, χ^2^(101) = 259.624, *p* < 0.001, yielding a *X*^2^/*df* ratio of 2.571; RMSEA = 0.052 (95%CI: 0.044, 0.060); CFI = 0.960, and TLI = 0.946. After the performed CFA, only Item 23 was excluded due to poor loading (< 0.40). All other items had a standardized factor loading estimate of ≥ 0.40, further indicating sufficient fit. The final resulting CFA produced three factors: Inattention (Items 12, 24, 25, 26, 27 and 29), Hyperactivity (Items 1, 14, 20 and 33) and Impulsivity (Items 3, 4, 7, 13, 17, 38, and 42), see Table 3 for the final factors.

**Table 3:** DAFRS items and subscales following confirmatory factor analysis.

Items (DAFRS)	Subscale
24. Has difficulties with learning, because he/she does not pay attention	*IA*
26. Performs poorly on tasks that require a lot of thinking	*IA*
25. When your dog is asked to perform a task, he/she is reluctant to comply or withdraws from the situation	*IA*
27. Your dog has difficulties solving tasks (i.e., makes many mistakes) that it is familiar with and has practised a lot before	*IA*
29. Quickly loses interest	*IA*
12. Has difficulties concentrating	*IA*
7. Has no self-control	*I*
38. Is difficult to control and handle	*I*
4. Once your dog "gets going", it is difficult to hold him/her back or stop	*I*
17. Is difficult to calm down	*I*
3. Becomes very excited when facing a new, mildly stressful situation (e.g., facing a new situation/place or meeting new people/dogs)	*I*
42. Reacts rashly to new stimuli, without considering the consequences	*I*
13. Is excessive, unrestrained, rampant	*I*
33. Would always play and run	*H*
14. Is rarely calm, even in familiar places, calm situations (e.g., at home)	*H*
20. Active even after fatiguing or hard exercise/ work	*H*
1. Fidgets, bustles	*H*

DAFRS Dog ADHD and Functionality Rating Scale, Owner-report form. Subscale Exploratory factor analysis and confirmatory factor analyses indicated categorisation of items to subscales (*IA* inattention, *H* hyperactivity, *I* Impulsivity).

The internal consistency measures were executed on the full dataset (*N* = 1168). The inattention subscale with six items had good internal consistency, *α* = 0.82. Hyperactivity subscale demonstrated good internal consistency *α* = 0.79. The third scale, impulsivity had an excellent internal consistency, *α* = 0.91.

**Figure 1:**
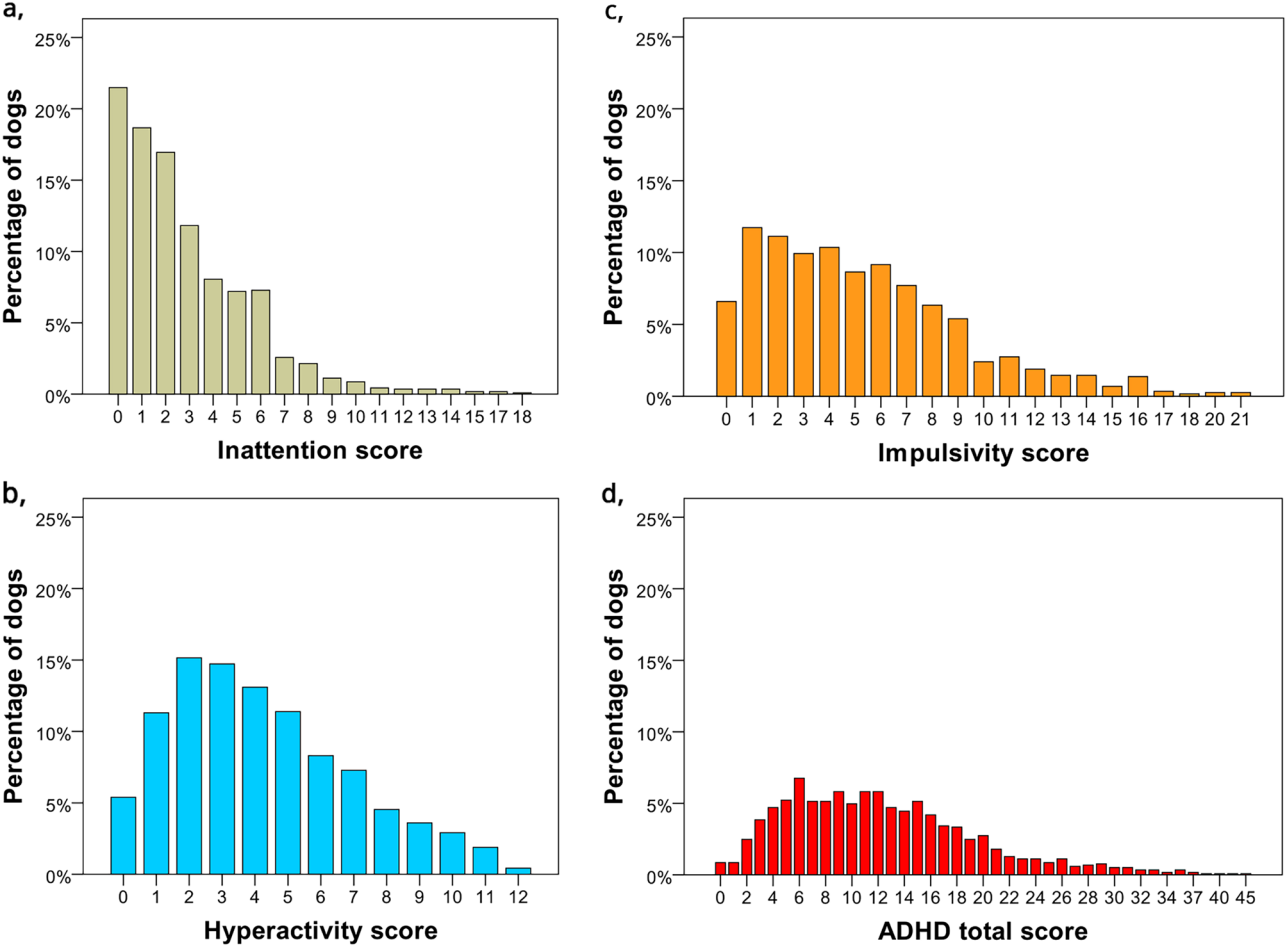
Distributions of subscale total scores for inattention (**a**), hyperactivity (**b**), impulsivity (**c**) and distribution of ADHD total scores (**d**) (*N* = 1168). *Note* The range of subscale scores after the final factor structure: inattention: 0–18 points, hyperactivity: 0–12 points, impulsivity: 0–21 points, ADHD total score: 0–51 points.

Response distributions for the ADHD subscale total scores (inattention, hyperactivity, impulsivity) and for the ADHD total scores are presented in Fig. 1. Mean item ratings (e.g., on average, how many respondents choose the given rating category) for the three ADHD subscales are also calculated and presented (see supplement, Fig. S1).

### Aim 4—Examine test–retest reliability of the DAFRS total score, subscale scores, and individual item scores

To examine test–retest reliability, we compared the owner ratings of dogs that we collected with the DAFRS by sending out the questionnaire to owners later again. The DAFRS demonstrated strong agreement between the two questionnaire completions regarding all ADHD factors: inattention (ICC = 0.845; 95% Cis = [0.799; 0.880], *p* < 0.001), hyperactivity (ICC = 0.885; 95% Cis = [0.851; 0.911], *p* < 0.001) and impulsivity (ICC = 0.902; 95% Cis = [0.873; 0.924], *p* < 0.001), and total ADHD scores (ICC = 0.904; 95% Cis = [0.875; 0.926], *p* < 0.001), meaning excellent test–retest reliability.

### Aim 5—Collect data from owners and trainers on the DAFRS

Regarding the agreement on inattention scale between owners and trainers, ICCs represented only fair agreement between raters (ICC = 0.485; 95% Cis = [0.181; 0.678], *p* = 0.003). Higher agreement was found examining the hyperactivity subscale (ICC = 0.660; 95% Cis = [0.453; 0.789], *p* < 0.001), meaning moderate agreement between the raters. Impulsivity score comparison resulted in moderate agreement between the raters (ICC = 0.682; 95% Cis = [0.488; 0.802], *p* < 0.001). Comparing the ADHD total scores resulted in moderate agreement between the raters (ICC = 0.624; 95% Cis = [0.394; 0.767], *p* < 0.001).

Distributions for the ADHD subscale total scores (inattention, hyperactivity, impulsivity) and for the ADHD total scores for owner and trainer ratings are presented in the supplement, as Fig. S2.

### Aim 6—Examine evidence of the convergent validity of the DAFRS: differences across age, sexes, and associations with functional impairment

**Figure 2:**
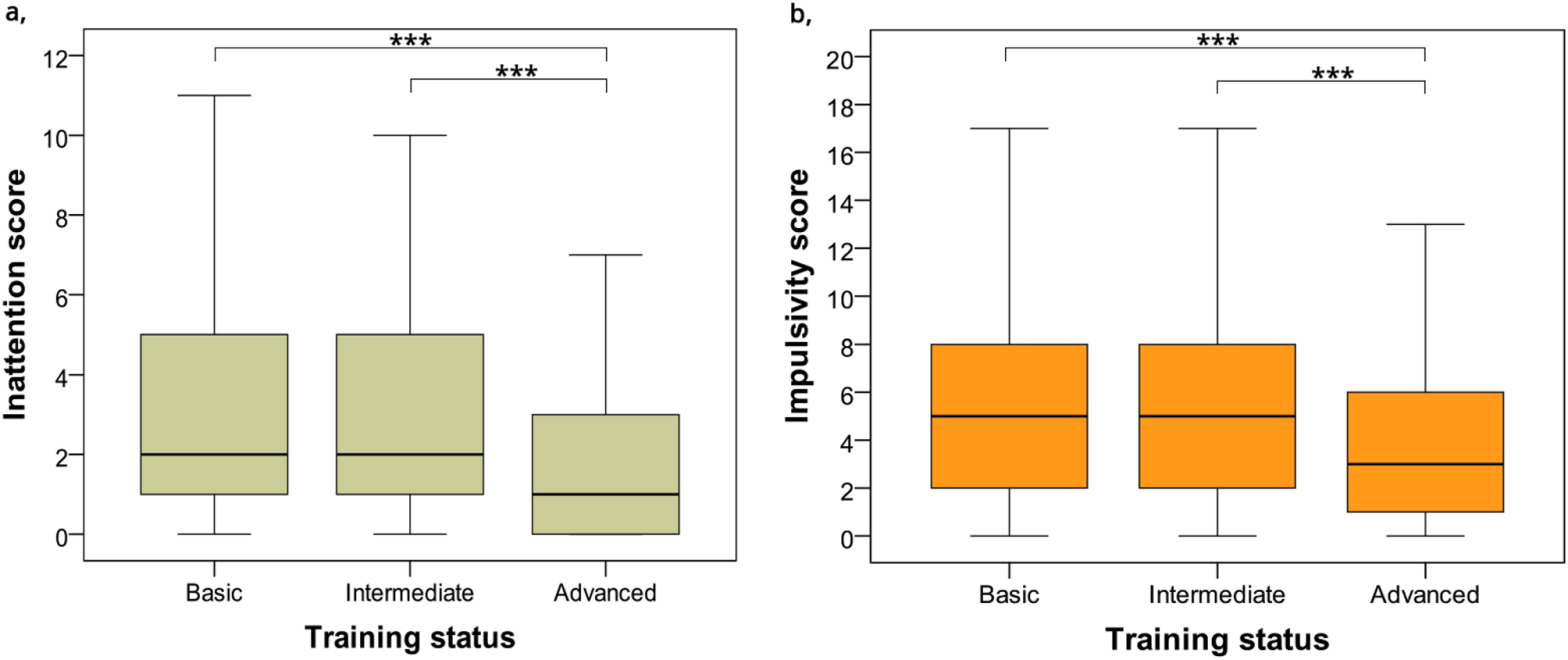
The association between training status and inattention (**a**) and impulsivity (**b**). *Note* We applied generalised linear mixed models with backward elimination, ‘*’ indicate significant differences (***p < 0.001).

Summarizing results on associations with inattention score and dogs’ sex, neutering status, age and training status, we found that sex (χ^2^_(1)_ = 0.390, *p* = 0.532) and the interaction between sex and neutering status were unrelated to inattention scores (χ^2^_(1)_ = 0.294, *p* = 0.588). Neutering status was associated with inattention (χ^2^_(1)_ = 6.054, *p* = 0.014), neutered dogs had higher inattention scores than intact dogs (*p* = 0.012, [0.09; 0.74]; *M*neutered = 2.93, *M*intact = 2.56). Age was not associated with inattention (χ^2^_(1)_ = 1.233, *p* = 0.267). Training status was associated with inattention (χ^2^_(1)_ = 31.689, *p* < 0.001), with post hoc tests indicating higher inattention scores for basic training compared to advanced training (*p* < 0.001, [0.70; 1.70]) and higher inattention scores for intermediate compared to advanced training (*p* < 0.001, [0.57; 1.54]), but no difference between dogs with basic training and intermediate training p = 0.876, [− 0.36; 0.64]); *M*basic = 3.07, *M*intermediate = 2.97, *M*advanced = 1.88; see Fig. 2a.

**Figure 3:**
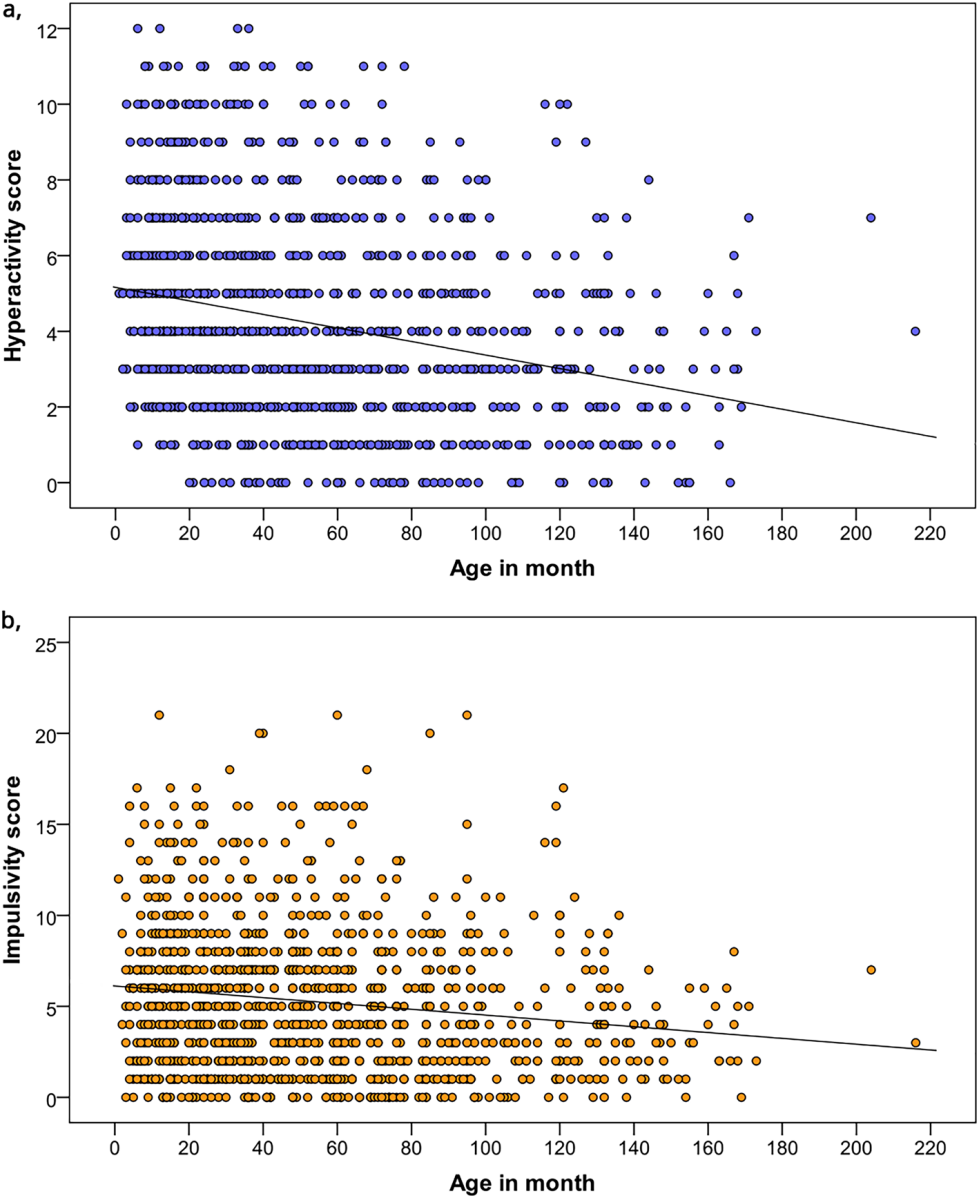
Association between age and hyperactivity (**a**) and impulsivity (**b**) scores. *Note* The coloured circles represent subjects.

Regarding associations with hyperactivity score, sex (χ^2^_(1)_ = 0.529, *p* = 0.467) and the interaction between sex and neutering status were unrelated to hyperactivity scores (χ^2^_(1)_ = 2.497, *p* = 0.114). Neutering status (χ^2^_(1)_ = 3.719, *p* = 0.054) was not associated with hyperactivity. Age was associated with hyperactivity (χ^2^_(1)_ = 58.104, *p* < 0.001); see Fig. 3a. Training status (χ^2^_(1)_ = 1.379, *p* = 0.502) was not associated with hyperactivity.

With regard to associations with impulsivity score, sex (χ^2^_(1)_ = 3.919, *p* = 0.048) was associated with impulsivity, males had higher impulsivity scores compared to females (*p* = 0.048, [0.00; 0.91]); *M*males = 5.49, *M*females = 5.02.

The interaction between sex and neutering status were unrelated to impulsivity scores (χ^2^_(1)_ = 0.263, *p* = 0.608).

Neutering was associated with impulsivity (χ^2^_(1)_ = 11.927, *p* < 0.001), neutered dogs had higher impulsivity scores than intact dogs (*p* < 0.001, [0.37; 1.32]; *M*neutered = 5.48, *M*intact = 4.88).

Age was associated with impulsivity (χ^2^_(1)_ = 28.537, *p* < 0.001); see Fig. 3b.

Training status was associated with impulsivity (χ^2^_(1)_ = 14.629, *p* < 0.001), with post hoc tests indicating higher impulsivity scores for basic training compared to advanced training (*p* < 0.001, [0.34; 1.78]) and higher impulsivity scores for intermediate compared to advanced training (*p* < 0.001, [0.40; 1.85]), but no difference between dogs with basic training and intermediate training (*p* = 0.993, [− 0.74; 0.60]); *M*basic = 5.34, *M*intermediate = 5.67, *M*advanced = 4.24; see Fig. 2b.

Regarding associations with functionality-inattention, sex (χ^2^_(1)_ = 22.276, *p* < 0.001) was associated with functionality-inattention, males had higher functionality-inattention scores compared to females (*p* < 0.001, [0.42; 1.03]; *M*males = 3.07, *M*females = 2.23).

The interaction between sex and neutering status were unrelated to functionality-inattention scores (χ^2^_(1)_ = 0.260, *p* = 0.610).

Neutering was associated with functionality-inattention (χ^2^_(1)_ = 7.971, *p* = 0.004), neutered dogs had higher functionality-inattention scores than intact dogs (*p* = 0.004, [0.15; 0.77]; *M*neutered = 2.70, *M*intact = 2.51).

Age was associated with functionality-inattention (χ^2^_(1)_ = 21.252, *p* < 0.001).

Training status was associated with functionality-inattention (χ^2^_(1)_ = 33.337, *p* < 0.001), with post hoc tests indicating higher functionality-inattention scores for basic training compared to advanced training (*p* < 0.001, [0.76; 1.71]) and higher functionality-inattention scores for intermediate compared to advanced training (*p* < 0.001, [0.41; 1.32]), but no difference between dogs with basic training and intermediate training (*p* = 0.184, [0.11; − 0.84]); *M*basic = 3.00, *M*intermediate = 2.74, *M*advanced = 1.70.

Considering the associations with functionality-hyperactivity, sex (χ^2^_(1)_ = 6.850, *p* = 0.009) was associated with functionality-hyperactivity, males had higher functionality-hyperactivity scores compared to females (*p* = 0.009, [0.12; 0.86]; *M*males = 3.07, *M*females = 2.49).

Neutering (χ^2^_(1)_ = 0.339, p = 0.561) and the interaction between sex and neutering status were unrelated to functionality-hyperactivity scores (χ^2^_(1)_ = 1.010, *p* = 0.315).

Age was associated with functionality-hyperactivity (χ^2^_(1)_ = 38.943, *p* < 0.001).

Training status was associated with functionality-hyperactivity (χ^2^_(1)_ = 16.570, *p* < 0.001), indicating higher functionality-hyperactivity scores for basic training compared to advanced training (*p* < 0.001, [0.46; 1.66]), but no difference between dogs with intermediate and advanced training (*p* = 0.052, [0.00; 1.13]) and with basic training and intermediate training (*p* = 0.092, [− 0.06; 1.05]); *M*basic = 3.17, *M*intermediate = 2.75, *M*advanced = 1.99.

Regarding associations with functionality-impulsivity, sex (χ^2^_(1)_ = 15.220, *p* < 0.001) was associated with functionality-impulsivity, males had higher functionality-impulsivity scores compared to females (*p* < 0.001, [0.42; 1.29]; *M*males = 3.89, *M*females = 2.94). The interaction between sex and neutering status were unrelated to functionality-impulsivity scores (χ^2^_(1)_ = 0.090, *p* = 0.764). Neutering was associated with functionality-impulsivity (χ^2^_(1)_ = 10.974, *p* < 0.001), neutered dogs had higher functionality-impulsivity scores than intact dogs (*p* < 0.001, [0.33; 1.22]; *M*neutered = 3.60, *M*intact = 3.07).

Age was associated with functionality-impulsivity (χ^2^_(1)_ = 13.420, *p* < 0.001).

Training status was associated with functionality-impulsivity (χ^2^_(1)_ = 20.602, *p* < 0.001), indicating higher functionality-impulsivity scores for basic training compared to advanced training (*p* < 0.001, [0.50; 1.82]) and higher functionality-impulsivity scores for intermediate training compared to advanced training (*p* < 0.001, [0.62; 1.97]), but no difference between dogs with basic training and intermediate training (*p* = 0.943, [− 0.81; 0.53]); *M*basic = 3.51, *M*intermediate = 3.85, *M*advanced = 2.30.

As for the relationships with vocalisation score, sex (χ^2^_(1)_ = 6.085, *p* = 0.014) was associated with vocalisation, males had higher vocalisation scores compared to females (*p* = 0.014, [0.04; 0.39]; *M*males = 0.92, *M*females = 0.72).

The interaction between sex and neutering status were unrelated to vocalisation scores (χ^2^_(1)_ = 0.020, *p* = 0.887). Neutering was associated with vocalisation (χ^2^_(1)_ = 5.142, *p* = 0.023), neutered dogs had higher vocalisation scores than intact dogs (*p* = 0.020, [− 0.38; − 0.03]; *M*neutered = 0.89, *M*intact = 0.71).

Age was not associated with vocalisation (χ^2^_(1)_ = 0.294, *p* = 0.588).

Training status was not associated with vocalisation (χ^2^_(1)_ = 4.887, *p* = 0.087).

Regarding associations with aggression score, sex (χ^2^_(1)_ = 5.059, *p* = 0.024) was associated with aggression, males had higher aggression scores compared to females (*p* = 0.026, [0.05; 0.78]; *M*males = 2.35, *M*females = 1.93).

Neutering (χ^2^_(1)_ = 1.321, *p* = 0.250), and the interaction between sex and neutering status were unrelated to aggression scores (χ^2^_(1)_ = 0.153, *p* = 0.696).

Age was not associated with aggression scores (χ^2^_(1)_ = 2.586, *p* = 0.108).

Training status was associated with aggression (χ^2^_(1)_ = 8.025, *p* = 0.018), with post hoc tests indicating higher aggression scores for intermediate training compared to advanced training (*p* = 0.019, [0.09; 1.28]), but no difference between dogs with basic training and intermediate training (*p* = 0.134, [− 0.97; 0.09]) and with basic training and advanced training (*p* = 0.662, [− 0.32; 0.80]); *M*basic = 2.02, *M*intermediate = 2.44, *M*advanced = 1.74.

**Figure 4:**
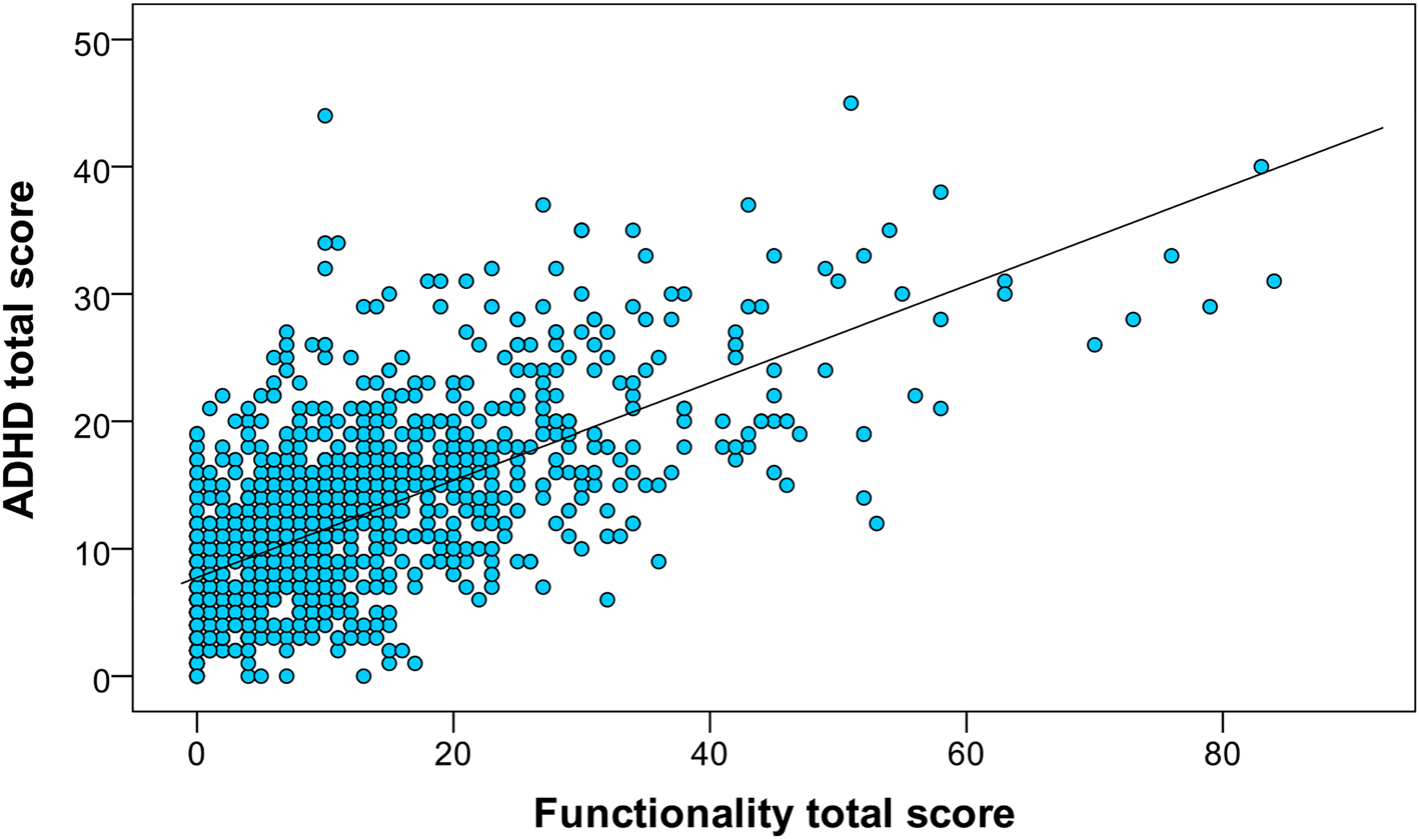
The relationship between ADHD total score and Functionality total score. *Note* The coloured circles represent subjects.

We examined the associations between ADHD subscale scores and functional impairment scores, and we found that all ADHD subscale scores are significantly positively correlated with the relevant Functionality subscale scores (Table 4). Inattention, Hyperactivity, Impulsivity and Aggression moderately correlated. All of the other variables correlated strongly (0.420 ≤ *r* ≤ 0.634), the correlation between ADHD total score and Functionality total score had the strongest correlation (*r* = 0.634, *p* < 0.001); see Fig. 4.

**Table 4:** Spearman’s correlations of ADHD subscale scores and functional impairment scores.

	Partial corr	r	*p* value
Inattention score—Functionality-inattention score	Training status	0.387	< 0.001
Hyperactivity score—Functionality-hyperactivity score	Age	0.371	< 0.001
Impulsivity score—Functionality-impulsivity score	Age	0.594	< 0.001
ADHD total score—Functionality total score	Age	0.634	< 0.001
Impulsivity score—Vocalisation score	Age	0.420	< 0.001
Impulsivity score—Aggression score	Age	0.398	< 0.001

## Discussion

With the aim of developing and assessing a novel, human-analogue questionnaire to measure inattention, hyperactivity and impulsivity in family dogs, we have successfully created a reliable and valid tool, the Dog ADHD and Functionality Rating Scale. We modified and supplemented previous canine ADHD assessment methods; we incorporated questions concerning functional impairments and combined owner ratings with expert ratings, similarly to the human ADHD assessments where teacher ratings complement parent ratings.

In contrast to the Dog ARS^32^, we did not translate a human ADHD questionnaire and adapted items to be applicable to dogs but generated conceptually- and experience-informed items. In addition, contrary to available rating scales on dog ADHD-like behaviours^32,33,56^, before exploratory factor analyses, we identified and removed ambiguous items. Owners were more confident in completing items about functionality than items describing a narrower context, so none of the former had to be excluded. The majority of the excluded specific items were reverse-worded (negations can be problematic as they require additional mental steps during processing^57^) or were constructed to assess inattention. Characteristics that are less accessible to an external observer, such as inattention, can be more difficult to recognise than characteristics that have more obvious behavioural manifestations (hyperactivity and impulsivity)^50,58^.

The determination of which questions are initially included in such a questionnaire inherently involves a degree of subjectivity and arbitrariness. However, it is crucial to note that our questionnaire items were chosen based either on established, previously validated questionnaire measures on dogs’ inattention, hyperactivity, and impulsivity^32,33^ or on the input of a panel of experts. It was imperative for the questionnaire to maintain a degree of brevity, thus, the factor analyses were employed to objectively identify and exclude questions from the questionnaire.

Moreover, it is vital to consider that dogs are subject to a highly diverse range of rearing environments. Unlike children, dogs do not undergo uniform, formal education, and not all dogs attend dog school. Therefore, an educational or school context, which is a common basis for human questionnaire measurements, can be applied to dogs in a much more limited capacity.

Contrary to factor analyses of the Dog ARS indicating two ADHD subscales, inattention and hyperactivity-impulsivity were found^32^ and three subscales with two of those mixed (inattention, hyperactivity/impulsivity-1, and hyperactivity/impulsivity-2)^56^, in the DAFRS, hyperactivity and impulsivity emerged as separate factors, as in human ADHD assessments^49,59,60^. By including additional impulsivity items, hyperactivity and impulsivity could be separated into two distinct factors. In humans, although it is accepted that hyperactivity and impulsivity are closely related, but differently manifesting traits^47^, the extent to which they are distinguishable depends on measurement method and the number of items^20,61^. Accordingly, that we were able to statistically differentiate hyperactivity and impulsivity may be the result of inclusion of more items that allow for an assessment of impulsivity that better reflects the heterogeneity of the behavioural manifestations of the phenomenon. So far, the Dog ARS comprised four impulsivity related items, some of them containing multiple statements, resulting in response uncertainty^28^. Among the Dog ARS items, there are few questions about self-control and/or behavioural-regulation, and the sudden reactions and excitement—as characteristics of impulsivity—were present but only in an above mentioned, multiple statement form (e.g., item 11: “My dog is likely to react hastily and that is why it is failing tasks.” and item 13: “My dog cannot wait as it has no self-control.”). Although hyperactivity and impulsivity share motor components, in our study, items describing activity and motoric components of behaviour belonged to the hyperactivity factor (i.e., “fidgets, bustles”), while impulsivity was more characterized by becoming excited and/or reacting rashly to new stimuli, not considering the consequences of behaviour. Moreover, our questionnaire contains several items which are related to behavioural regulation, and lack of self-control, similarly to the Dog Impulsivity Rating Scale, where behavioural regulation and responsiveness appear as independent factors^33^. Although dogs exhibited natural variation in impulsivity, and previous studies indicate that certain phenomena associated with impulsivity in humans can be also explored in dogs (e.g., behavioural disinhibition^26,27^ and intolerance to delayed rewards^62–64^), there is currently limited understanding of the regulatory mechanisms associated with impulsivity in dogs or the extent to which it is comparable to humans (where the exact characteristics of these mechanisms are also debated^10,65,66^).

In addition to inattention, hyperactivity, and impulsivity, a fourth factor, “Vocalisation” emerged in our analysis. Even though we did not retain it as a separate factor—as it contained only two items—, we recommend including these as functionality items, as excessive vocalisation is likely related to ADHD (here, vocalisation items were correlated with impulsivity). The fact that symptoms of human ADHD include verbal impulsivity and these symptoms are also evaluated during the diagnostic process (i.e., "talks excessively," "blurts out answers," and "interrupts or intrudes others") emphasises the retention of these items. Even in humans, some ADHD dimension models reported separable factors for verbal and motor components of hyperactivity and impulsivity^45^. Although symptoms of verbal impulsivity might be challenging or impossible to adopt in a dog questionnaire.

All subscales exhibited acceptable or better internal consistency. The observed distributions of the three ADHD subscale scores and ADHD total score were skewed towards lower values (“J-shaped”), which is very similar to distributions in human populations^67–69^. The distribution of ADHD scores in the general population is not expected to follow a normal distribution because by definition, psychopathology is designated by deviance from the average and infrequency^70^. Scaling is a further important similarity between the DAFRS and human questionnaires; the typical item rating on the DAFRS was “Never” or “Sometimes”, which is remarkably similar to data with humans, where the average rating for the general (non-clinical) population is between “Never” and “Sometimes”^70^. The DAFRS questionnaire, including its subscales, displayed excellent test–retest reliability.

In contrast to the original form of the Dog ARS^32^, in our earlier study, we introduced the idea of involving dog trainers as experts in the evaluation process^28^, similar to human assessment methods. In line with findings on the Dog ARS^28^, the comparison of owner and trainer ratings revealed fair agreement for the inattention and moderate agreement for the hyperactivity and impulsivity subscales, which is consistent with data obtained with humans^58^. These findings may reflect differences in the extent to which the manifestations of these characteristics are accessible to observes, i.e., as discussed previously, compared to inattention, hyperactivity and impulsivity are relatively easier to judge for an observer.

In children, one reason for low agreement between parents and teachers may be attributable to differences in environmental demands, e.g., at school, children are more likely to be engaged in tasks that require sustained attention^71^. Low agreement may also be due to contextual variation in the manifestations of symptoms across different settings (i.e., home vs. school). Indeed, data from multiple informants are expected to be only weakly to moderately correlated, with each rater providing unique and valid information^50^. The same arguments can be made about the herein observed low and moderate agreement between owners and trainers, where owners and trainers observe the dog in different contexts (i.e., home vs. dog school). Further, owners may be more biased and also have a smaller comparison pool^72^. Relative to the entire sample, in the subsample of dogs with trainer ratings, distribution of owner ratings was less skewed. Thus, assessing dogs with training experience (and trainer ratings) may be more similar to assessing children, and such dogs may be more suitable for modelling ADHD.

DAFRS showed convergent validity in terms of associations with age, sex, neutering and training status and ADHD factor and impairment scores. Prior data were mixed on differences across sexes, indicating higher scores in males^31^ but also no differences across sexes^28,32,33,56^ in inattention and hyperactivity/impulsivity scores. In humans, boys exhibit more (or more apparent, and disruptive) hyperactive/impulsive symptoms, and are more often referred to clinicians^73^.

Here, aggression and vocalisation scores were higher in males, in line with prior findings showing aggression (for a review, see Scandurra et al.^74^) and behavioural problems are more common in male dogs^53,75,76^.

Across current and earlier studies, neutered dogs has higher inattention^56^, impulsivity^77^, vocalisation, and impairment scores^53,78^. It is possible that (some) dogs were neutered because of behavioural problems and impulsivity^79^. Alternatively, although shelter dogs are generally neutered prior to adoption^80^, shelter conditions favour development of unwanted behaviours^81^ and many dogs may have been relinquished because of behaviour problems^82,83^. Additionally, it is also plausible that the owners’ decisions on neutering were primarily driven by health or practical considerations. As we did not collect information on what motivated the owners to neuter their dogs, thus further studies are necessary to disentangle these explanations.

Our findings of a negative association between age and hyperactivity and impulsivity are consistent with data obtained with humans^17^, though relations between age and ADHD symptoms in dogs are mixed, with some indicating a negative association with inattention only^32^ and others showing a negative association with both inattention and hyperactivity/impulsivity^31,56^.

Training status negatively correlated with inattention and impulsivity, but not with hyperactivity. Others have found training status was negatively associated with inattention but not with hyperactivity/impulsivity^32,35^ but also that training status was negatively correlated with both inattention and hyperactivity/impulsivity^28,56^. In our study, differentiation between hyperactivity and impulsivity may account for the differential associations of training status with these traits. As hyperactivity and impulsivity had always been combined it was not possible to determine what drives associations. It is important to note that the lack of training, limited daily exercise, and a lack of engagement in activities that fulfil the dog’s needs may enhance inattentive, hyperactive, and impulsive behaviours^31^, similarly to observations on children^84,85^.

Indicating evidence of convergent validity, ADHD subscale scores were positively associated with relevant functionality scores (e.g., inattention score with functionality-inattention score). The strongest correlation was found between ADHD total score and functionality total score, suggesting that extreme manifestations of ADHD traits are strongly associated with functional deficits in dogs. Our results are in line with previous studies showing that inattentive and hyperactive/impulsive dogs are more prone to show behavioural problems, such as repetitive behaviour^31^, fear-, compulsion- and separation-related behaviours^54^. Notably, we revealed a positive correlation between impulsivity and aggression, a well-established link in human research^86^.

The challenges associated with measuring human ADHD and the inherent limitations to questionnaire-based assessment methods are relevant to our study. Notably, some of the items of the DAFRS can potentially allude to diverse underlying factors. For instance, even in typically behaving dogs, a high score on a particular item can be attributed. Additionally, other conditions and behavioural problems, such as anxiety and fear may lead to elevated scores on certain items. It is important to highlight that a similar phenomenon exists in human ADHD diagnostics, particularly when assessing symptoms by questionnaires, such as that the symptoms of ADHD and anxiety can often overlap^87^, as ADHD and anxiety disorders are highly comorbid in humans^87–89^. In humans, it remains uncertain whether anxiety arises as a consequence of ADHD symptoms, and the role of environmental factors in symptomatology is yet to be definitively established^89^. Distinguishing these behaviours and symptoms in dogs may also pose a significant challenge, emphasizing the future importance of differential diagnosis.

Our study has limitations, such as the mean time interval between the test–retest (92 days) and owner-trainer evaluations (28 days) could allow for changes in dogs’ behaviour to some extent. Intensive training or changes in the dogs’ environment during this period might influence dogs’ behaviour and thus our results.

As the examination of the dog as a model of human ADHD is a relatively novel approach, it is important to note that the DAFRS may not cover every behaviour, which might have importance in ADHD-like behaviours in dogs (e.g., excessive chewing and destruction, lack of satiety, difficulties in communication with humans and other dogs^42^).

Although the use of questionnaires can be a valid and effective tool, the responses can be burdened by subjectivity. Certain ADHD-like behaviours and aggression might be subject to social desirability bias^90,91^, leading to a more positive portrayal of the behaviours, and potential underestimation of their severity^92^. Considering functionality-related behavioural problems, and the externalizing nature of ADHD symptoms^93^, subjectivity exists in how these symptoms are perceived and evaluated by the parent of the child in humans^52^, but this may be also true for dog owners. Thus, the extent to which a dog’s behaviour disrupts everyday life of the owner may rely on individual perspectives and interpretations of the owner. The owners’ assessment may also depend on their experience in breed differences and in dog behaviour (e.g., whether they had a dog before or attended dog school). Moreover, as in all dog studies, participating owners cannot be considered a representative sample because they must be particularly interested in the behaviour of their dogs as they are willing to take the time to fill out the questionnaire. Further measures are needed to assess the external validity of the owners’ reports, especially applying simple behavioural tests to assess ADHD dimensions.

It must be emphasised that in applications of the Dog ARS so far^26,27^, results have always been obtained with typical dog populations, not with dogs with ADHD. The Dog ARS is not appropriate for diagnosis or screening, as ADHD characteristics themselves are valid indices of ADHD only if they are functionally impairing. Although impaired functioning is a diagnostic criterion for human ADHD, until now the standard and systematic measurement of functionality has received limited attention in ADHD assessments in dogs^94^. We revealed that functional deficits are positively connected to ADHD traits measured by our questionnaire, which suggests that dogs with extreme phenotypes of ADHD traits present functional impairments.

In conclusion, our findings support the validity of our novel human-analogue questionnaire as a tool for measuring family dogs’ inattention, hyperactivity, impulsivity and relevant functional impairments based on owner/trainer ratings. Apart from measuring the variation in ADHD dimensions within a normal population, this tool may provide an opportunity to examine whether ADHD can be diagnosed in dogs based on the criteria established in the human literature. This includes systematic, standard assessment of expert ratings and functionality. Further research is required to explore the extent of analogies between human ADHD and canine ADHD-like behaviours, and to evaluate whether extreme scores on different subscales accompanied by relevant functional deficits demonstrate behavioural pathology in dogs.

## Methods

### Ethics statement

All procedures were carried out in accordance with relevant guidelines and regulations for human participants (dog owners and dog trainers) as volunteers participating in the study. The study was carried out in accordance with the Declaration of Helsinki and approved by the Hungarian Ethics Committee of “United Ethical Review Committee for Research in Psychology (EPKEB)” (reference number of approval: EPKEB-202304). Owners and trainers gave informed consent to participate in the online questionnaire study.

### Subjects

Participants were recruited through the Department of Ethology (Eötvös Loránd University in Budapest, Hungary) participant pool and website, popular social networking sites, and via snowball sampling. Dog trainers were recruited by the owners (who were asked to invite their dog’s trainer to participate in the study). Sample sizes differed across research questions (Table 1). For data on demographics of the different samples, see the supplementary material, appendix A.

### Procedures

We present results on factor analysis and report results on internal consistency, test–retest reliability, and inter-rater agreement and the results of construct and convergent validity. Our methods for the sub-aims are summarised in Table 1.

#### Procedure for aim 1—Compile a new questionnaire: Dog ADHD and Functionality Rating Scale (DAFRS)

The questionnaire was developed on the basis of two validated dog rating scales, the Dog ADHD Rating Scale (Dog ARS)^32^ and the Dog Impulsivity Assessment Scale (DIAS)^33^ and we also formulated novel items to better assess inattention, hyperactivity and impulsivity (appendix B contains information on which rating scale served as a basis for particular items). The questionnaire was constructed in association with a clinical expert and researcher with vast experience of ADHD and associated problems in humans, further, the initial form of the questionnaire and the entire development process were reviewed and discussed with dog behaviour expert veterinarians, including a diplomat from ECAWBM and a European Veterinary Specialist in Behavioural Medicine. Other experts—ethologists, human ADHD clinicians and researchers, and dog trainers—also took part in the development of the questionnaire. To demonstrate face validity, we employed the Delphi method as it is a systematic procedure that is commonly used in human questionnaire research to reach a consensus among a chosen group of experts, especially when investigating or defining areas characterised by significant uncertainty and/or a lack of agreed knowledge^95^. According to this method, to include all possible behaviours in the questionnaire that may be connected to ADHD characteristics in dogs, we asked the expert panel to review and comment on the initial form of the questionnaire. Review and discussion of the initial form of the questionnaire was aimed to determine whether the questionnaire covered all aspects of the domains intended to be measured and contained no irrelevant items or items that might refer to other conditions. The experts’ comments were reviewed by the authors, who reached consensus concerning rephrasing, changing of the order of items, clarifications, adding items or a reduction in the number of items of the originally formulated pool of items. We followed evidence-based guidelines throughout the rating scale development process and analyses according to modern human ADHD research^96–98^ and to validated dog personality and temperament measurements^99,100^. Aim 1 analysis sample consisted of *N* = 1168 dogs (see details in supplement, appendix A). We collected data from 1087 dog owners, each of whom evaluated a single dog, and 81 owners provided evaluations for multiple dogs, with one owner evaluated 5 dogs, 5 owners evaluated 3 dogs, and 75 owners evaluated 2 dogs each.

Based on our earlier replication study on the Dog ARS^28^, we modified, split or rephrased the items that owners reported to be hard to answer and where results showed a high proportion of IDK answers (appendix B). Specifically, three items were identified earlier with a high IDK response rate earlier in the Dog ARS using this method: item 10 “My/this dog solves simple tasks easily, but often has difficulties with complicated tasks, even those are known and have been often practised.”, item 11: “My/this dog is likely to react hastily and that is why it is failing tasks.”, and item 13: “My/this dog cannot wait as it has no self-control.”.

We added additional questions on activity and impulsivity (especially on behavioural impulsivity) so that hyperactivity and impulsivity are better distinguishable, as in human ADHD rating scales^49,59,60^. We adopted items from the Dog Impulsivity Assessment Scale (DIAS) in order to improve conceptual coverage of impulsivity in dogs^33^ (appendix B). We further included questions from human ADHD questionnaires, especially focusing on impulsivity questions, rewrote or transformed them in a way that they are more applicable to dogs^101–103^.

Overall, 42 items were formulated to assess inattention (15 items), hyperactivity (14 items) and impulsivity (13 items), with 29 items adopted from other measures, and 13 newly formulated (see appendix B). The dog owners were asked to rate how often their dogs behaved according to the statements formulated in items. Consistent with most human ADHD questionnaires^104^, a 4-point Likert-type response format scale was used to assess the frequency of behaviours consistent with inattention, hyperactivity, and impulsivity: 0 = never, 1 = sometimes, 2 = often, and 3 = very often. Items of inattention, activity and impulsivity were presented in a mixed order. The questionnaire includes reverse-worded items (inattention vs attention) (see appendix C in supplement) that were reverse-scored for analyses. Final factor scores are obtained by summing individual item scores of that factor: inattention (6 items, range: 0–18 points), hyperactivity (4 items, range: 0–12 points) and impulsivity (7 items, range: 0–21 points) and the ADHD total score is obtained by summing all individual items (17 items, range: 0–51 points) (also see supplement, appendix D). Questionnaire completion took 10–15 min on average.

As the questionnaire was administered to a Hungarian sample, items were formulated in Hungarian. However, so that findings can be reported and the questionnaire used in cross-cultural research, items were translated into English by a native speaker.

Commonly used human questionnaires^105–107^ were reviewed to identify items assessing functionality, and identified items were adapted for applicability to dogs.

Given their relevance to ADHD and to functioning, we included items on impairments that could stem from inattention, hyperactivity, and impulsivity (e.g., “*For your dog, to what extent can the following problems (if any) be attributed to the dog’s impulsivity? [Disturbs acquaintances, guests, e.g., jumps up, pinches]*”) and on aggression^108–110^. In terms of comparability with human assessments, in the case of children, it is much easier to ask about behaviours that can be relevant as symptoms of ADHD and occur in educational settings. Since not all dogs receive training or attend dog school, it is unfeasible to exclusively focus on training situations for measurement purposes. In contrast, nearly all dogs are routinely walked by their owners which constitute a more common context where ADHD-like behaviours may manifest. Consequently, some questions related to this context were incorporated as a compromise. Moreover, in humans, ADHD –as an externalising disorder– is characterized by behaviours that are outwardly directed and these behaviours may impact far more heavily upon others than upon the individual with the condition^93^. In this regard, behaviours which are difficult to bear with and disruptive to the dog’s social environment might be worth to measure, as these behaviours might have importance in measurement of ADHD-like behaviours and related functionality problems in dogs. Based on this, we included such items in the functionality assessment (e.g., “*Other dog owners don’t like walking with us*”). We formed 7 impairment questions per trait (inattention, hyperactivity, impulsivity and aggression) that were similarly worded but with the relevant ADHD characteristic varied, e.g., scale and scoring for impairment due to hyperactivity: 0 = there is no such a problem; 0 = it is a problem but not as a result of excessive activity, 1 = it is a problem, and, to some extent, is a result of excessive activity; 2 = it is a problem, and, more or less, is a result of excessive activity; 3 = it is a problem, and is largely a result of excessive activity. We assigned zero point for the first two categories as we were interested in whether impairments and problematic behaviours presented due to inattention, hyperactivity, impulsivity, and/or aggression; the presence of a problem was deemed irrelevant if it was not connected to these phenomena.

The following results are necessary for appropriate description of subsequent analyses and methods: Two items (Item 8: Cannot be quiet, whines or barks a lot even when there is nothing special to evoke this; and Item 30: If your dog starts to bark or whine, it is difficult to silence him/her) that were originally included as ADHD items but not in the functionality assessment, formed a separate factor, “Vocalisation” after the exploratory factor analyses of ADHD items (Aim 3). Following evidence-based guidelines for scale development^55^, we eliminated these two items from the ADHD items, but we decided to include them in the functionality items. For these two items, a 4-point Likert-type response format scale was applied (0 = never, 1 = sometimes, 2 = often, and 3 = very often).

Finally, functionality-inattention, functionality-hyperactivity, functionality-impulsivity, aggression and vocalisation scores were calculated (i.e., added together) from the above-mentioned items, and these together gave the overall functionality total score, with 30 items on functionality (appendix D).

#### Procedure for aim 2—Identify and eliminate ambiguous items/functionality items in the DAFRS

To identify ambiguous questions, the DAFRS was modified via inclusion of an IDK response option for each question (DAFRS IDK). Guidelines on questionnaire development suggests testing the IDK response option in a pretest on a separate sample and simplifying, redesigning, or omitting questions with high IDK response rates from the final questionnaire^97,111^. Further, adding an IDK response option to items in the final form of the questionnaire may impact item stability and reduce the overall questionnaire score since IDK responses are treated as missing data. This could potentially result in total scores that do not accurately reflect dogs’ inattention, hyperactivity, and impulsivity levels, which could affect subsequent analyses and comparisons. Following this and assuming that respondents select the IDK option when a question is difficult to respond to or they are uncertain in their rating^97^, higher IDK response counts indicate items that are problematic and need to be dropped before further analyses.

Aim 2 analysis sample consisted of *N* = 210 dogs for the DAFRS IDK (independent sample from the *N* = 1168 sample, see Table 1 and sample details in supplement, appendix A). Items with an IDK response rate higher than 5% were considered to be ambiguous. These items were dropped before factor analysis.

#### Procedure for aim 3—Examine the factor structure—including to determine whether hyperactivity can be distinguished from impulsivity in dogs—and the internal consistency of the DAFRS

As our third aim was to explore the factor structure of the DAFRS, we used both exploratory and confirmatory factor analyses to reveal factor structure. Additionally, as a part of validation, we aimed to calculate the reliability of the resulting factors.

For the factor analyses, we used the data collected from 1168 dog owners (Aim 1). For exploratory factor analyses, we randomly selected one half of the data (*n* = 584), and we performed confirmatory factor analyses on the second half (*n* = 584), as these two analyses must be done using separate data sets^112^.

To address the aim of establishing the factor structure of the DAFRS, we conducted an exploratory factor analysis (EFA) on a randomly selected half of the participants’ data (*n* = 584). To examine the number and content of the latent variables that represent items included in the DAFRS, procedures consistent with evidence-based guidelines for scale development^96^ were followed. EFA was run in the R statistical environment version 3.6.0. using RStudio^113^. To determine the structure of the questionnaire items EFA was conducted (principal axis factoring function with oblimin rotation) on the remaining 36 items out of 42 regarding inattention, activity and impulsivity, after dropping out those items which had IDK response rate higher than 5% (Aim 2). As our items had four response categories, we decided to use a polychoric correlation matrix on our data to avoid the effects of items with asymmetric distributions^114^. The model was cleaned using systematic elimination of low loading variables (< 0.4 on any subscale) or sat on multiple components with comparable absolute loadings (> 0.4 on two or more subscales). To determine factor retention, we examined eigenvalues above 1 and the “elbow” in the Scree Plot^115^. The number of extracted components were re-determined in each cycle with parallel analysis (paran). After deletion of an item, EFA was re-run until no items were indicated for deletion. Once the factor structure had been finalised, we conducted a confirmatory factor analysis on the remaining half of the data (*n* = 584). CFA was conducted in IBM SPSS AMOS 24.0.0. Both modification indices and the items’ estimated standardized factor loadings were referenced. Model fit was examined using the *X*^2^/*df* ratio, root mean square error of approximation (RMSEA), the comparative fit index (CFI), and the Tucker-Lewis index (TLI). Conventionally, a *X*^2^/*df* ratio of 5:1^116,117^, a RMSEA ≤ 0.10, a CFI and TLI ≥ 0.90 indicate sufficient fit^118^ and *X*^2^/*df* ratio of 2, RMSEA ≤ 0.06, and CFI and TLI ≥ 0.95 indicate excellent fit^119^. To test reliability (Cronbach’s alpha) and present normative data, we calculated reliability estimates for each of the factors on the full sample (*N* = 1168) and calculated means and standard deviations for each item. Additionally, each subscale was assessed for evidence of internal consistency (considered acceptable if α > 0.70^120^). Distributions for subscale scores, ADHD total score and mean ratings for subscale items were also presented.

#### Procedure for aim 4—Examine the test–retest reliability of the DAFRS

To assess test–retest reliability of the DAFRS, the developed questionnaire (Aim 3; *N* = 1168) was mailed again within a short time interval to participants who provided their email addresses for further cooperation in the present study. Intraclass correlation coefficients were calculated for the final subscales and the total score (Aim 3).

Test–retest reliability analysis was performed based on questionnaires returned from the entire sample (*n* = 231/1168). The mean test–retest interval was 92 days, with a range of 1–231 days (*SD* = 56.73). Aim 4 analysis sample consisted of 231 dogs (see details in supplement, appendix A).

Intraclass correlation coefficients (ICC) with corresponding 95% Cis were computed for measuring test–retest reliability of the DAFRS. ICCs represent the ratio of between-subjects variance to total variance and are the appropriate metric (as opposed to Pearson’s *r*) for assessing test–retest reliability when observations are not independent^121^. Specifically, in SPSS, the ICC model was a two-way mixed model with estimates for absolute agreement and 95% Cis. In accordance with convention, ICCs, which range from − 1 to 1 were interpreted as follows: 0–0.2 as poor, 0.3–0.4 as fair, 0.5–0.6 as moderate, 0.7–0.8 as strong, and > 0.8 as almost perfect^122^. Of note, it is possible for ICCs to be negative when the within-group variance exceeds the between-groups variance, suggesting a measure is not reliable.

#### Procedure for aim 5—Collect data from owners and trainers on the DAFRS

Similarly to the human ADHD evaluation process, where multi-informant reports of symptoms are considered in diagnosis, we aimed to include expert ratings (dog trainers) of the dogs’ behaviour and compare them to the owner ratings. We asked owners who had been working together with a dog trainer to send out a link for a separate questionnaire (see appendix E) for their dog’ trainers, thus we could collect expert ratings for the dogs.

Both for dog owners and dog trainers, the DAFRS IDK form (Aim 2) was used to have comparable ratings, because dog trainers may not face every situation described in the questionnaire with the particular dog.

We could use *n* = 70 dogs’ data for measuring inter-rater agreement between the dog trainers and owners, because both respondents had to fill out the DAFRS + IDK questionnaire. We collected data from 19 dog trainers for 70 dogs. The mean time difference between the two questionnaire fills by the two evaluators were 28 days, with a range of 0 to 132 days (*SD* = 33.55). Aim 5 analysis sample consisted of 70 dogs (see details in supplement, appendix A).

Questionnaire for trainers included all items from the developed questionnaire, regarding inattention, activity and impulsivity, with adding an IDK option to all items (Aim 2). We only included in the present analysis the questions that both the owner and the trainer had to answer (questions on attention, activity and impulsivity, see appendix E in supplement).

Intraclass correlation coefficients were used to compare dog trainer and owner ratings for the same dog (scores for inattention, hyperactivity, impulsivity, ADHD total). Owner and trainer rating distributions of subscale total scores and of ADHD total scores were also presented.

#### Procedure for aim 6—Examine evidence of the convergent validity of the DAFRS: differences across age, sexes, and associations with functional impairment

So far, age has mostly been used to assess the Dog ARS external validity^28^, but given the complex relationship between ADHD and age, this approach is overly simplistic^17,123^. Although the manifestations of ADHD symptoms change with age, the behavioural problems and functional impairments associated with the disorder (although also manifested differently) persist during development^17,123^. As functionality is a cardinal component of the diagnosis, we opine that it is much more meaningful to use functionality in addition to age to examine external validity. We also included sex and neutering status in our analyses, as ADHD is more prevalent in boys than girls^124^ and the manifestation of symptoms might be different regarding gender^125^.

As a test for external validity, we examined the links among (1) sex, neutering status, age, training status and ADHD factor scores and functional impairment scores, and links among (2) ADHD factor scores and functional impairment scores. The final DAFRS subscales (following confirmatory factor analysis) formed the basis to calculate the ADHD factor scores (inattention, hyperactivity, impulsivity and ADHD total score).

Owners reported the birth date of their dog, or if they did not know they reported the estimated age. Age for 8 dogs were not reported in the questionnaire, thus the final sample contained data for 1160 dogs out of 1168.

The interaction between sex and neutering status, and the associations of sex, neutering status, age and training status with the dependent variables (i.e., inattention, hyperactivity, impulsivity, functionality-inattention, functionality-hyperactivity, functionality-impulsivity, vocalisation and aggression), were examined in generalised linear mixed models with backward elimination. Age was entered as a covariate, sex, neutering and training status as fixed factors and subject as random factor. Following backward elimination, variables were removed in order of decreasing significance, starting with the interactions, until only significant variables were in the model. Tweedie with log link option was used as model type, given that the dependent variables had zero scores and skewed distributions, resulting in non-normal distribution of residuals. Sidak correction was applied to account for multiple comparisons. Assumptions were considered prior to all analyses, these were met.

As age and/or training might have an effect on all the examined variables (that showed non-normal distribution), to analyse the links among ADHD factor scores and functional impairment scores, Spearman’s partial rank correlations were performed whilst controlling for age or training status (we used age where age was associated with the given variable or training status where age did not have an effect, but training status was associated with the given variable), with a written syntax in SPSS. To control for multiple comparisons, Benjamini–Hochberg correction was conducted and results based on the adjusted *p* values are reported.

### Supplementary Information

Supplementary Information.

## Data Availability

The datasets generated and analysed during the current study are available in the Figshare repository, using the following link: https://doi.org/10.6084/m9.figshare.22806404.v1.
