# Supplementary material for: Development of a human-analogue, 3-symptom domain Dog ADHD and Functionality Rating Scale (DAFRS)
Source: Sci Rep. 2024 Jan 20;14:1808. doi: 10.1038/s41598-024-51924-9 (PMC10799898; doi:10.1038/s41598-024-51924-9)
Supplement: Supplementary file 1 — Supplementary Information. [file 41598_2024_51924_MOESM1_ESM.docx]

**Supplementary material to:**

**Development of a human-analogue, 3-symptom domain Dog ADHD and Functionality Rating Scale (DAFRS)**

**Barbara Csibra ^1,2^ *, Nóra Bunford ^3^ and Márta Gácsi ^1,4^**

^1^ Eötvös Loránd University, Institute of Biology, Department of Ethology, Pázmány Péter sétány 1/C, Budapest, 1117 Hungary;

^2^ Doctoral School of Biology, Institute of Biology, ELTE Eötvös Loránd University, Pázmány Péter sétány 1/C, Budapest, 1117 Hungary

^3^ Clinical and Developmental Neuropsychology Research Group, Research Centre for Natural Sciences, Institute of Cognitive Neuroscience and Psychology, Magyar tudósok körútja 2, Budapest, 1117 Hungary;

^4^ MTA-ELTE Comparative Ethology Research Group, Pázmány Péter sétány 1/C, Budapest, 1117 Hungary;

***** Correspondence: csibrabarbara@gmail.com

**Appendix A**

Information on sample demographics for the different samples. Sample sizes differed across research questions and were accordingly indicated separately below for each question.

*1. Aim 1 – Compile a new questionnaire: Dog ADHD and Functionality Rating Scale (DAFRS).*

*1. a Clarify ambiguous items revealed in Csibra et al. (2022) in the Dog ARS (Vas et al., 2007)*

The sample and information are based on the Csibra et al. 2022 publication (Aim 3/ Question 1 and Question 2), sampling was performed by Csibra and colleagues in our earlier study (Csibra et al. 2022). The Dog ARS IDK questionnaire was used for this sample, the Dog ARS IDK-O: Dog ARS with “I don’t know” option for owners (N = 520; *M*age=55.01 months, *SD*=39.59), and the Dog ARS IDK-T: Dog ARS with “I don’t know” option for trainers (N = 86; *M*age=44.31 months, *SD*=28.96).

*1. b Formulate additional items that allow for separate assessment of impulsivity*

The sample consisted of 1168 dogs: 553 male (249 intact males, 304 neutered males) and 615 female (210 intact females, 405 spayed females) dogs (*M*age=54.21 months, *SD*=38.82).

The distribution of the breeds in the sample:

341 mixed breed dogs,; 1 Afghan hound, 5 Airedale terrier, 3 akita inu, 3 Alaskan malamute, 3 American akita, 1 American bulldog, 11 American pitbull terrier, 20 American Staffordshire terrier, 3 greyhound, 1 bulldog, 19 English cocker spaniel, 3 English pointer, 1 English setter, 3 Argentin dog, 5 Australian shepherd, 1 Bavarian mountain hound, 2 basset hound, 7 beagle, 1 bearded collie, 2 Beauceron, 4 Groenendael, 19 Malinois, 3 Tervuren, 11 Bernese mountain dog, 4 bichon Frise, 2 bichon Havanese, 3 biewer terrier, 2 Bordeaux dog, 82 border collie, 2 border terrier, 3 Boston terrier, 8 boxer, 1 fila Brasileiro, 7 briard, 1 bullmastiff, 11 bullterrier, 1 cairn terrier, 7 cane corso, 1 Catahoula leopard dog, 4 cavalier King Charles spaniel, 1 coton de Tulear, 4 Czechoslovakian wolfdog, 10 Chihuahua, 1 Dalmatian, 9 Dobermann, 6 fox terrier (smooth-haired), 1 fox terrier (wire-haired), 7 French bulldog, 28 golden retriever, 15 Havanese, 1 Hokkaido, 2 Dutch shepherd dog, 2 Hovawart, 5 Irish setter, 14 Jack Russell terrier, 4 Canary mastiff, 2 Anatolian shepherd dog, 2 Caucasian shepherd, 3 Chinese crested, 1 komondor, 6 standard schnauzer, 7 medium poodle, 4 Central Asian shepherd, 1 Portuguese podengo, 3 kuvasz, 46 Labrador retriever, 2 lagotto romagnolo, 1 Lancashire heeler, 4 landseer, 1 Leonberger, 13 Hungarian greyhound, 35 vizsla, 4 Maltese, 4 pug, 2 Moscow watchdog, 12 mudi, 7 great Dane, 68 German shepherd, 1 German miniature spitz, 2 German medium spitz, 2 German pinscher, 2 German pointer (wire-haired), 8 German pointer (short-haired), 1 Italian greyhound, 13 giant schnauzer, 4 standard poodle, 1 borzoi, 1 black Russian terrier, 1 papillon, 1 parson Russel terrier, 6 Pekingese, 1 xoloitzcuintli, 5 great Pyrenees, 2 Pomeranian, 1 Portuguese water dog, 9 puli, 5 pumi, 10 Rottweiler, 2 Sarplaninac, 1 Chinese shar-pei, 1 Shetland sheepdog, 4 shiba inu, 1 shih-tzu, 2 sinka, 10 collie (rough), 1 Scottish terrier, 4 Staffordshire bull terrier, 13 white Swiss shepherd dog, 7 Siberian husky, 3 dachshund (wire-haired), 2 dachshund (long-haired), 15 dachshund (short-haired), 3 Tibetan terrier, 1 toy poodle, 4 miniature pinscher, 6 miniature schnauzer, 1 miniature spitz, 1 miniature dachshund (short-haired), 1 miniature dachshund (wire-haired), 11 miniature poodle, 1 Newfoundland, 1 Irish red and white setter, 2 Weimaraner, 1 Pembroke Welsh corgi, 4 West highland white terrier, 20 whippet, 13 Yorkshire terrier.

*1. c Add items that allow for assessment of functional impairment*

For this research question, the same sample was used as in Aim1/1.b.

*Aim 2 – Identify and eliminate ambiguous items/functionality items in the DAFRS.*

The sample consisted of 210 dogs: 106 male (55 intact males, 51 neutered males) and 104 female (29 intact females, 75 spayed females) dogs (*M*age=53.30 months, *SD*=39.94).

The distribution of the breeds in the sample:

57 mixed breed dogs; 2 Airedale terrier, 1 akita inu, 1 American pitbull terrier, 2 American Staffordshire terrier, 5 English cocker spaniel, 1 Argentin dog, 1 Australian shepherd, 1 Australian kelpie, 1 beagle, 1 Beauceron, 2 Malinois, 1 Tervuren, 1 bichon Frise, 1 bichon Havanese, 1 bichon Bolognese, 11 border collie, 2 boxer, 4 bullterrier, 1 cane corso, 3 cavalier King Charles spaniel, 1 Czechoslovakian wolfdog, 3 chihuahua, 1 Dalmatian, 9 Dobermann, 1 fox terrier (smooth-haired), 2 fox terrier (wire-haired), 5 French bulldog, 10 golden retriever, 1 Gordon setter, 1 Hanoverian scenthound, 3 Havanese, 1 Hovawart, 1 Jack Russell terrier, 1 medium poodle, 1 Labrador retriever, 1 lagotto romagnolo, 13 vizsla, 3 Maltese, 2 pug, 1 Moscow watchdog, 7 mudi, 15 German shepherd, 2 German miniature spitz, 1 German medium spitz, 1 German pinscher, 2 German pointer (short-haired), 1 giant schnauzer, 1 standard poodle, 1 parson Russel terrier, 3 pumi, 2 Rottweiler, 1 Shetland sheepdog, 2 shih-tzu, 1 Staffordshire bull terrier, 1 Siberian husky, 2 dachshund (wire-haired), 5 dachshund (short-haired), 1 miniature pinscher, 1 miniature schnauzer, 2 miniature dachshund (short-haired), 2 miniature poodle, 1 Welsh terrier, 1 Yorkshire terrier.

*Aim 3 – Examine the factor structure – including to determine whether hyperactivity can be distinguished from impulsivity in dogs – and the internal consistency of the DAFRS.*

For this research question, the same sample was used as in Aim1/1.b.

*Aim 4 – Examine test-retest reliability of the DAFRS total score, subscale scores, and individual item scores.*

This sample is a subsample of the DAFRS sample (*n*=231/1168, see Aim 1/1.b). We included those dogs, where the owners filled out the DAFRS again within a short time interval, for test-retest analysis. The mean test-retest interval was 92 days.

The sample consisted of 231 dogs: 90 male (38 intact males, 52 neutered males) and 141 female (41 intact females, 100 spayed females) dogs (*M*age=61.50 months, *SD*=56.22).

The distribution of the breeds in the sample:

88 mixed breed dogs; 1 Afghan hound, 1 akita inu, 1 American akita, 2 American Staffordshire terrier, 3 Argentin dog, 2 Australian shepherd, 1 beagle, 1 Malinois, 1 Bernese mountain dog, 1 bichon Havanese, 14 border collie, 1 Boston terrier, 1 boxer, 1 bullterrier, 1 cane corso, 1 cavalier King Charles Spaniel, 1 Czechoslovakian wolfdog, 1 Chihuahua, 1 Dalmatian, 2 fox terrier (smooth-haired), 1 fox terrier (wire-haired), 2 French bulldog, 7 golden retriever, 2 Havanese, 1 Dutch shepherd, 1 Irish setter, 3 Jack Russell terrier, 2 Caucasian shepherd dog, 1 standard schnauzer, 2 medium poodle, 1 Central Asian shepherd, 9 Labrador retriever, 2 lagotto romagnolo, 1 Leonberger, 1 Hungarian greyhound, 10 vizsla, 1 Maltese, 1 Moscow watchdog, 4 mudi, 1 great Dane, 14 German shepherd, 1 German pointer (short-haired), 4 giant schnauzer, 1 parson Russel terrier, 3 puli, 2 Rottweiler, 1 Sarplaninac, 1 Shetland shepherd dog, 2 collie (rough), 1 Scottish terrier, 2 white Swiss shepherd dog, 1 dachshund (wire-haired), 4 dachshund (short-haired), 1 toy poodle, 1 miniature schnauzer, 1 miniature dachshund (short-haired), 4 miniature poodle, 1 Newfoundland, 1 Pembroke Welsh corgi, 2 west highland white terrier, 2 whippet, 2 Yorkshire terrier.

*Aim 5 – Collect data from owners and trainers on the DAFRS.*

We could use N = 70 dogs’ data for measuring inter-rater agreement between the dog trainers and owners, because both respondents had to fill out the DAFRS+IDK questionnaire. We collected data from 19 dog trainers for 70 dogs. The mean time difference between the two questionnaire fills by the two evaluators were 28 days, with a range of 0 to 132 days (SD = 33.55).

The sample consisted of 70 dogs: 39 male (15 intact males, 24 neutered males) and 31 female (6 intact females, 25 spayed females) dogs (*M*age=42.90 months, *SD*=43.67).

The distribution of the breeds in the sample:

19 mixed breed dogs; 1 American Staffordshire terrier, 1 English cocker spaniel, 1 beagle, 15 border collie, 1 bullterrier, 1 Chihuahua, 4 golden retriever, 1 Irish setter, 1 lagotto romagnolo, 3 Maltese, 3 mudi, 1 great Dane, 4 German shepherd, 1 German miniature spitz, 1 Pomeranian, 1 shiba inu, 1 white Swiss shepherd dog, 4 dachshund (short-haired), 3 Tibetan terrier, 2 miniature poodle, 1 west highland white terrier.

*Aim 6 – Examine evidence of the convergent validity of the DAFRS: Differences across age, sexes, and associations with functional impairment*

For this research question, the same sample was used as in Aim1/1.b.

**Appendix B**

The developed attention, activity and impulsivity-related items for the Dog ADHD and Functionality Rating Scale (DAFRS). The questionnaire was developed on the basis of two validated dog rating scales, the Dog ADHD Rating Scale (Dog ARS) (Vas et al. 2007) and the Dog Impulsivity Assessment Scale (DIAS) (Wright et al. 2011). (Aim1/1.a-b.). The "Basis" column indicates which items are based on the Dog ARS or the DIAS. Q: questions; R: reverse-worded and reverse-scored items; Cat. = a priori categorisation of items to scales; IA = inattention; H = hyperactivity; I = Impulsivity.

| QUESTIONS ON ATTENTION, ACTIVITY, IMPULSIVITY | | | |
| --- | --- | --- | --- |
| Q/R | Cat. | Item description | Basis |
|  |  | How typical are the following statements of your dog? |  |
| 1 | H | Fidgets, bustles. | Dog ARS |
| 2 | H | Gets too revved up, acts as if "driven by a motor". |  |
| 3 | I | Becomes very excited when facing a new, mildly stressful situation (e.g., facing new situation/place or meeting new people/dogs). | DIAS |
| 4 | H | Once your dog "gets going", it is difficult to hold him/her back or stop. | Dog ARS |
| 5 | I | Interrupts you, is intrusive. | DIAS |
| 6 | IA | You have to repeat your requests (e.g., commands) several times, as your dog does not appear to pay attention. |  |
| 7 | I | Has no self-control. | Dog ARS |
| 8 | H | Cannot be quiet, whines or barks a lot even when there is nothing special to evoke this. | Dog ARS |
| 9 R | IA | Completes simple tasks easily, even many times in a row. | Dog ARS, DIAS |
| 10 | I | Bites without forewarning (e.g., tensing up, growling). | DIAS |
| 11 | I | Becomes very excited during greetings (e.g., shows extreme reactions: whining, urinating, excessive jumping) | DIAS |
| 12 | IA | Has difficulties concentrating. | Dog ARS |
| 13 | H | Is excessive, unrestrained, rampant. | Dog ARS |
| 14 | H | Is rarely calm, even in familiar places, calm situations (e.g., at home). |  |
| 15 | I | Does not think before he/she acts. | DIAS |
| 16 | I | Has difficulty waiting, is impatient. | Dog ARS, DIAS |
| 17 | H | Is difficult to calm down. | DIAS |
| 18 R | IA | Watches you (follows you with its gaze) while you are engaging in daily activities. |  |
| 19 | IA | Your dog is mentally scattered. |  |
| 20 | H | Active even after fatiguing or hard exercise/ work. |  |
| 21 | H | Has difficulty maintaining "stay". | Dog ARS |
| 22 R | IA | It is easy to keep his/her attention for long periods of time. |  |
| 23 | H | Sleeps little, is active even during the night. |  |
| 24 | IA | Has difficulties with learning, because he/she does not pay attention. | Dog ARS, DIAS |
| 25 | H | When your dog is asked to perform a task, he/she is reluctant to comply or withdraws from the situation. |  |
| 26 | IA | Performs poorly on tasks that require a lot of thinking. | Dog ARS |
| 27 | IA | Has difficulties (makes many mistakes) with completing complicated tasks, even if he/she is familiar with the task and has frequently practiced he/she before. | Dog ARS |
| 28 | I | Tends to wolf down his/her food. |  |
| 29 | IA | Quickly loses intertest. | Dog ARS, DIAS |
| 30 | H | If your dog starts to bark or whine, it is difficult to silence him/her. | Dog ARS |
| 31 | I | Is persistent, he/she does not give up easily. | DIAS |
| 32 | I | Tends to behave hastily and that is why he/she makes mistakes in tasks. | Dog ARS |
| 33 | H | Would always play and run. | Dog ARS |
| 34 | IA | Loses its toys while playing and forgets to search for them (if not instructed to do so). |  |
| 35 | I | Seems to get excited for no reason. | DIAS |
| 36 | IA | Is easily distracted by extraneous stimuli (e.g., sounds, moving objects, other dogs/people). | Dog ARS |
| 37 | I | Continues to do something even if he/she knows he/she will be told off or get punished. | DIAS |
| 38 | H | Is difficult to control and handle. | Dog ARS |
| 39 R | IA | It is easy to attract his/her attention. | Dog ARS |
| 40 R | IA | Seems to be contemplative, thoughtful. |  |
| 41 | IA | Seems to not pay attention to you, even when he/she knows you are speaking directly to him/her. | Dog ARS |
| 42 | I | Reacts rashly to new stimuli, without considering the consequences. | Dog ARS, DIAS |

**Appendix C**

The original form of the Dog ADHD and Functionality Rating Scale (DAFRS), before factor analyses (Aim1/1.a-b.). Space was provided for alternative answers where necessary. Asterisk-marked questions are not mandatory to be answered. Q: questions; R: reverse-worded and reverse-scored items; Cat. = a priori categorisation of items to scales; IA = inattention; H = hyperactivity; I = Impulsivity. Functionality item categorisation by the related impairments: F-IA = functionality-inattention; F-H: functionality-hyperactivity; F-I = functionality-impulsivity; F-A = functionality-aggression.

Responses to items indicated in the table below with “R” need to be reverse-scored: “(0) Never – (1) Rarely – (2) Often – (3) Very often” need to be reversed to “(3) Never – (2) Rarely – (1) Often – (0) Very often”.

| Description of the questionnaire for owners | | | |
| --- | --- | --- | --- |
| Dear Owner! The main goal of our research is to develop a new questionnaire to measure dogs' level of attention, activity, and impulsivity. Completing the questionnaire takes approximately 15 minutes, and we hope you will find it interesting! There are no right or wrong answers, but please, answer all questions to the best of your knowledge! Our goal is to get the most accurate picture of your dog. Please complete the questionnaire for one dog at a time. If you want to complete it for additional dogs, please re-open the questionnaire and submit a new response. Data are stored for the sole purpose of communication regarding participation in research and are not communicated to any third parties. Thank you for helping us! | | | |
| Consent statement and privacy policy | | | |
| After data are processed and analysed, results will be published in the form of scientific presentations, papers, or educational materials. During data processing, data are stored at the Department of Ethology at Eötvös Loránd University. Research participants are informed about results upon reasonable request. Participation in this research is voluntary and anonymous, and the data obtained is used for scientific purposes only. In compliance with relevant data protection laws, personally identifying data are treated confidentially and stored separately from the rest of research data. Data sets shared with other researchers or made publicly available do not contain identifying information. Research data are further handled as part of a large database. Should you have any questions or comments regarding this research, please, contact our research team: Barbara Csibra, PhD student, ELTE Department of Ethology, email: csibrabarbara@gmail.com | | | |
| Consent Statement: I have read the consent and privacy policy information and am aware of the goals of- and processes involved in the current research. By participating in this research, I consent to my anonymized data being shared and treated as part of a larger database. I am aware that I may discontinue participating in this research at any time. | | | Yes/No (The questionnaire is terminated if you answer "No.") |
| GENERAL QUESTIONS | | | |
| Q/R | Cat. | Item description | Scale and scoring |
| 1 | - | Owner's name (or unique identifier) (Data are stored for the sole purpose of communication regarding participation in research and are not communicated to any third parties.) | Text |
| 2 | - | Email address (It is used only for the communication necessary for the tests and is not passed on to third parties.) | Text |
| 3 | - | The dog's name | Text |
| 4 | - | The dog's date of birth in YYYY.MM.DD format. If you DO NOT KNOW your dog's exact birth date, please, enter the following numbers: "1212.12.12." and go to the next question where you can enter the estimated age of the dog. | Text |
| 5 | - | Your dog's estimated date of birth. If you don't know exactly when your dog was born, please estimate its age by following the options below. | Text |
| 6 | - | The dog's breed | Text |
| 7 | - | If you selected "other breed", please describe the breed here: * | Text |
| 8 | - | The dog's sex | male, female, neutered male, neutered female |
| 9 | - | What kind of training has your dog received? | (0) None or I trained him/her at home – (1) Basic obedience (dog-school/trainer) – (2) Higher obedience (dog-school) |
| QUESTIONS ON ATTENTION, ACTIVITY, IMPULSIVITY | | | |
|  |  | How typical are the following statements of your dog? |  |
| 1 | H | Fidgets, bustles. | (0) Never – (1) Rarely – (2) Often – (3) Very often |
| 2 | H | Gets too revved up, acts as if "driven by a motor". |  |
| 3 | I | Becomes very excited when facing a new, mildly stressful situation (e.g., facing new situation/place or meeting new people/dogs). |  |
| 4 | H | Once your dog "gets going", it is difficult to hold him/her back or stop. |  |
| 5 | I | Interrupts you, is intrusive. |  |
| 6 | IA | You have to repeat your requests (e.g., commands) several times, as your dog does not appear to pay attention. |  |
| 7 | I | Has no self-control. |  |
| 8 | H | Cannot be quiet, whines or barks a lot even when there is nothing special to evoke this. |  |
| 9 R | IA | Completes simple tasks easily, even many times in a row. |  |
| 10 | I | Bites without forewarning (e.g., tensing up, growling). |  |
| 11 | I | Becomes very excited during greetings (e.g., shows extreme reactions: whining, urinating, excessive jumping) |  |
| 12 | IA | Has difficulties concentrating. |  |
| 13 | H | Is excessive, unrestrained, rampant. |  |
| 14 | H | Is rarely calm, even in familiar places, calm situations (e.g., at home). |  |
| 15 | I | Does not think before he/she acts. |  |
| 16 | I | Has difficulty waiting, is impatient. |  |
| 17 | H | Is difficult to calm down. |  |
| 18 R | IA | Watches you (follows you with its gaze) while you are engaging in daily activities. |  |
| 19 | IA | Your dog is mentally scattered. |  |
| 20 | H | Active even after fatiguing or hard exercise/ work. |  |
| 21 | H | Has difficulty maintaining "stay". |  |
| 22 R | IA | It is easy to keep his/her attention for long periods of time. |  |
| 23 | H | Sleeps little, is active even during the night. |  |
| 24 | IA | Has difficulties with learning, because he/she does not pay attention. |  |
| 25 | H | When your dog is asked to perform a task, he/she is reluctant to comply or withdraws from the situation. |  |
| 26 | IA | Performs poorly on tasks that require a lot of thinking. |  |
| 27 | IA | Has difficulties (makes many mistakes) with completing complicated tasks, even if he/she is familiar with the task and has frequently practiced he/she before. |  |
| 28 | I | Tends to wolf down his/her food. |  |
| 29 | IA | Quickly loses intertest. |  |
| 30 | H | If your dog starts to bark or whine, it is difficult to silence him/her. |  |
| 31 | I | Is persistent, he/she does not give up easily. |  |
| 32 | I | Tends to behave hastily and that is why he/she makes mistakes in tasks. |  |
| 33 | H | Would always play and run. |  |
| 34 | IA | Loses its toys while playing and forgets to search for them (if not instructed to do so). |  |
| 35 | I | Seems to get excited for no reason. |  |
| 36 | IA | Is easily distracted by extraneous stimuli (e.g., sounds, moving objects, other dogs/people). |  |
| 37 | I | Continues to do something even if he/she knows he/she will be told off or get punished. |  |
| 38 | H | Is difficult to control and handle. |  |
| 39 R | IA | It is easy to attract his/her attention. |  |
| 40 R | IA | Seems to be contemplative, thoughtful. |  |
| 41 | IA | Seems to not pay attention to you, even when he/she knows you are speaking directly to him/her. |  |
| 42 | I | Reacts rashly to new stimuli, without considering the consequences. |  |
| QUESTIONS ON FUNCTIONALITY | | | |
|  |  | In the case of your dog, to what extent are the following problems (if they occur) attributable to the dog’s inattention? (For example, in the case of the first question, to what extent is your dog's inattention responsible for your concern about releasing it?) |  |
| 1 | F-IA | Cannot be released in an unfamiliar place | (0) There is no such problem –  (0) It is a problem, but not a result of inattention – (1) It is a problem, and, to some extent, is a result of inattention – (2) It is a problem, and, more or less, is a result of inattention – (3) It is a problem, and is largely a result of inattention |
| 2 | F-IA | Is unable to engage in activities with other dogs in a conflict-free manner, e.g., play with them, get to know them |  |
| 3 | F-IA | It learns with difficulty and slowly, as indicated, for example, by it lagging behind in dog school |  |
| 4 | F-IA | Other dog owners don't like walking with us |  |
| 5 | F-IA | Annoys, bothers guests and relatives, e.g., by jumping on them, nipping them |  |
| 6 | F-IA | I am concerned to release it in the presence of a toddler (I'm afraid it will knock the toddler over) |  |
| 7 | F-IA | It disrupts mobility in the home/garden |  |
|  |  | In the case of your dog, to what extent are the following problems (if they occur) attributable to the dog’s impulsivity? (For example, in the case of the first question, to what extent is your dog's impulsivity responsible for your concern about releasing it?) |  |
| 8 | F-I | Cannot be released in an unfamiliar place | (0) There is no such problem –  (0) It is a problem, but not a result of impulsivity – (1) It is a problem, and, to some extent, is a result of impulsivity – (2) It is a problem, and, more or less, is a result of impulsivity – (3) It is a problem, and is largely a result of impulsivity |
| 9 | F-I | Is unable to engage in activities with other dogs in a conflict-free manner, e.g., play with them, get to know them |  |
| 10 | F-I | It learns with difficulty and slowly, as indicated, for example, by it lagging behind in dog school |  |
| 11 | F-I | Other dog owners don't like walking with us |  |
| 12 | F-I | Annoys, bothers guests and relatives, e.g., by jumping on them, nipping them |  |
| 13 | F-I | I am concerned to release it in the presence of a toddler (I'm afraid it will knock the toddler over) |  |
| 14 | F-I | It disrupts mobility in the home/garden |  |
|  |  | In the case of your dog, to what extent are the following problems (if they occur) attributable to the dog’s excessive activity? (For example, in the case of the first question, to what extent is your dog's excessive activity responsible for your concern about releasing it?) |  |
| 15 | F-H | Cannot be released in an unfamiliar place | (0) There is no such problem –  (0) It is a problem, but not a result of excessive activity – (1) It is a problem, and, to some extent, is a result of excessive activity – (2) It is a problem, and, more or less, is a result of excessive activity – (3) It is a problem, and is largely a result of excessive activity |
| 16 | F-H | Is unable to engage in activities with other dogs in a conflict-free manner, e.g., play with them, get to know them |  |
| 17 | F-H | It learns with difficulty and slowly, as indicated, for example, by it lagging behind in dog school |  |
| 18 | F-H | Other dog owners don't like walking with us |  |
| 19 | F-H | Annoys, bothers guests and relatives, e.g., by jumping on them, nipping them |  |
| 20 | F-H | I am concerned to release it in the presence of a toddler (I'm afraid it will knock the toddler over) |  |
| 21 | F-H | It disrupts mobility in the home/garden |  |
|  |  | In the case of your dog, to what extent are the following problems (if they occur) attributable to the dog’s aggression? (For example, in the case of the first question, to what extent is your dog's aggression responsible for your concern about releasing it?) |  |
| 22 | F-A | Cannot be released in an unfamiliar place | (0) There is no such problem –  (0) It is a problem, but not a result of aggression – (1) It is a problem, and, to some extent, is a result of aggression – (2) It is a problem, and, more or less, is a result of aggression – (3) It is a problem, and is largely a result of aggression |
| 23 | F-A | Is unable to engage in activities with other dogs in a conflict-free manner, e.g., play with them, get to know them |  |
| 24 | F-A | It learns with difficulty and slowly, as indicated, for example, by it lagging behind in dog school |  |
| 25 | F-A | Other dog owners don't like walking with us |  |
| 26 | F-A | Annoys, bothers guests and relatives, e.g., by jumping on them, nipping them |  |
| 27 | F-A | I am concerned to release it in the presence of a toddler (I'm afraid it will knock the toddler over) |  |
| 28 | F-A | It disrupts mobility in the home/garden |  |

**Appendix D**

The final form of the Dog ADHD and Functionality Rating Scale (DAFRS), Owner-report form after factor analyses (Aim3). Space was provided for alternative answers where necessary. Asterisk-marked questions are not mandatory to be answered. Q: questions, R: reverse-worded and reverse-scored items; IA = inattention; H = hyperactivity; I = Impulsivity. Functionality item categorisation by the related impairments: F-IA = functionality-inattention; F-H: functionality-hyperactivity; F-I = functionality-impulsivity; F-A = functionality-aggression; F-V = functionality-vocalization. R = Reverse scored items.

Calculation of the scores

Responses to items indicated in the table below with “R” are to be reverse-scored: “(0) Never – (1) Rarely – (2) Often – (3) Very often” need to be reversed to “(3) Never – (2) Rarely – (1) Often – (0) Very often”.

The ADHD subscales after factor analyses are inattention (IA), hyperactivity (H) and impulsivity (I); where the score of the items belonging to the given factor are added together to get the factor total score. The ADHD total score is calculated by adding together the total scores of the three factors (IA total score + H total score + I total score). We calculated the following facet scores, where the scores related to the given facets are added to get a total score of Functionality: F-IA: functionality-inattention related items; F-H: functionality-hyperactivity related items; F-I: functionality-impulsivity related items; F-A: functionality-aggression related items; F-V: functionality-vocalization related items. The Functionality total score is calculated by adding the scores of the related facets (F-IA + F-H + F-I + F-A + F-V).

| Description of the questionnaire for owners | | | |
| --- | --- | --- | --- |
| Dear Owner! The main goal of our research is to develop a new questionnaire to measure dogs' level of attention, activity, and impulsivity. Completing the questionnaire takes approximately 15 minutes, and we hope you will find it interesting! There are no right or wrong answers, but please, answer all questions to the best of your knowledge! Our goal is to get the most accurate picture of your dog. Please complete the questionnaire for one dog at a time. If you want to complete it for additional dogs, please re-open the questionnaire and submit a new response. Data are stored for the sole purpose of communication regarding participation in research and are not communicated to any third parties. Thank you for helping us! | | | |
| Consent statement and privacy policy | | | |
| After data are processed and analysed, results will be published in the form of scientific presentations, papers, or educational materials. During data processing, data are stored at the Department of Ethology at Eötvös Loránd University. Research participants are informed about results upon reasonable request. Participation in this research is voluntary and anonymous, and the data obtained is used for scientific purposes only. In compliance with relevant data protection laws, personally identifying data are treated confidentially and stored separately from the rest of research data. Data sets shared with other researchers or made publicly available do not contain identifying information. Research data are further handled as part of a large database. Should you have any questions or comments regarding this research, please, contact our research team: Barbara Csibra, PhD student, ELTE Department of Ethology, email: csibrabarbara@gmail.com | | | |
| Consent Statement: I have read the consent and privacy policy information and am aware of the goals of- and processes involved in the current research. By participating in this research, I consent to my anonymized data being shared and treated as part of a larger database. I am aware that I may discontinue participating in this research at any time. | | | Yes/No (The questionnaire is terminated if you answer "No.") |
| GENERAL QUESTIONS | | | |
| Q/R | Subscale/Facet | Item description | Scale and scoring |
| 1 | - | Owner's name (or unique identifier) (Data are stored for the sole purpose of communication regarding participation in research and are not communicated to any third parties.) | Text |
| 2 | - | Email address (It is used only for the communication necessary for the tests and is not passed on to third parties.) | Text |
| 3 | - | The dog's name | Text |
| 4 | - | The dog's date of birth in YYYY.MM.DD format. If you DO NOT KNOW your dog's exact birth date, please, enter the following numbers: "1212.12.12." and go to the next question where you can enter the estimated age of the dog. | Text |
| 5 | - | Your dog's estimated date of birth. If you don't know exactly when your dog was born, please estimate its age by following the options below. | Text |
| 6 | - | The dog's breed | Text |
| 7 | - | If you selected "other breed", please describe the breed here: * | Text |
| 8 | - | The dog's sex | male, female, neutered male, neutered female |
| 9 | - | What kind of training has your dog received? | (0) None or I trained him/her at home – (1) Basic obedience (dog-school/trainer) – (2) Higher obedience (dog-school) |
| QUESTIONS ON ATTENTION, ACTIVITY, IMPULSIVITY | | | |
|  |  | How typical are the following statements of your dog? |  |
| 1 | H | Fidgets, bustles. | (0) Never – (1) Rarely – (2) Often – (3) Very often |
| 3 | I | Becomes very excited when facing a new, mildly stressful situation (e.g., facing new situation/place or meeting new people/dogs). |  |
| 4 | I | Once your dog "gets going", it is difficult to hold him/her back or stop. |  |
| 7 | I | Has no self-control. |  |
| 12 | IA | Has difficulties concentrating. |  |
| 13 | I | Is excessive, unrestrained, rampant. |  |
| 14 | H | Is rarely calm, even in familiar places, calm situations (e.g., at home). |  |
| 17 | I | Is difficult to calm down. |  |
| 20 | H | Active even after fatiguing or hard exercise/ work. |  |
| 24 | IA | Has difficulties with learning, because he/she does not pay attention. |  |
| 25 | IA | When your dog is asked to perform a task, he/she is reluctant to comply or withdraws from the situation. |  |
| 26 | IA | Performs poorly on tasks that require a lot of thinking. |  |
| 27 | IA | Has difficulties (makes many mistakes) with completing complicated tasks, even if he/she is familiar with the task and has frequently practiced he/she before. |  |
| 29 | IA | Quickly loses intertest. |  |
| 33 | H | Would always play and run. |  |
| 38 | I | Is difficult to control and handle. |  |
| 42 | I | Reacts rashly to new stimuli, without considering the consequences. |  |
| QUESTIONS ON FUNCTIONALITY | | | |
|  |  | In the case of your dog, to what extent are the following problems (if they occur) attributable to the dog’s inattention? (For example, in the case of the first question, to what extent is your dog's inattention responsible for your concern about releasing it?) |  |
| 1 | F-IA | Cannot be released in an unfamiliar place | (0) There is no such problem –  (0) It is a problem, but not a result of inattention – (1) It is a problem, and, to some extent, is a result of inattention – (2) It is a problem, and, more or less, is a result of inattention – (3) It is a problem, and is largely a result of inattention |
| 2 | F-IA | Is unable to engage in activities with other dogs in a conflict-free manner, e.g., play with them, get to know them |  |
| 3 | F-IA | It learns with difficulty and slowly, as indicated, for example, by it lagging behind in dog school |  |
| 4 | F-IA | Other dog owners don't like walking with us |  |
| 5 | F-IA | Annoys, bothers guests and relatives, e.g., by jumping on them, nipping them |  |
| 6 | F-IA | I am concerned to release it in the presence of a toddler (I'm afraid it will knock the toddler over) |  |
| 7 | F-IA | It disrupts mobility in the home/garden |  |
|  |  | In the case of your dog, to what extent are the following problems (if they occur) attributable to the dog’s impulsivity? (For example, in the case of the first question, to what extent is your dog's impulsivity responsible for your concern about releasing it?) |  |
| 8 | F-I | Cannot be released in an unfamiliar place | (0) There is no such problem –  (0) It is a problem, but not a result of impulsivity – (1) It is a problem, and, to some extent, is a result of impulsivity – (2) It is a problem, and, more or less, is a result of impulsivity – (3) It is a problem, and is largely a result of impulsivity |
| 9 | F-I | Is unable to engage in activities with other dogs in a conflict-free manner, e.g., play with them, get to know them |  |
| 10 | F-I | It learns with difficulty and slowly, as indicated, for example, by it lagging behind in dog school |  |
| 11 | F-I | Other dog owners don't like walking with us |  |
| 12 | F-I | Annoys, bothers guests and relatives, e.g., by jumping on them, nipping them |  |
| 13 | F-I | I am concerned to release it in the presence of a toddler (I'm afraid it will knock the toddler over) |  |
| 14 | F-I | It disrupts mobility in the home/garden |  |
|  |  | In the case of your dog, to what extent are the following problems (if they occur) attributable to the dog’s excessive activity? (For example, in the case of the first question, to what extent is your dog's excessive activity responsible for your concern about releasing it?) |  |
| 15 | F-H | Cannot be released in an unfamiliar place | (0) There is no such problem –  (0) It is a problem, but not a result of excessive activity – (1) It is a problem, and, to some extent, is a result of excessive activity – (2) It is a problem, and, more or less, is a result of excessive activity – (3) It is a problem, and is largely a result of excessive activity |
| 16 | F-H | Is unable to engage in activities with other dogs in a conflict-free manner, e.g., play with them, get to know them |  |
| 17 | F-H | It learns with difficulty and slowly, as indicated, for example, by it lagging behind in dog school |  |
| 18 | F-H | Other dog owners don't like walking with us |  |
| 19 | F-H | Annoys, bothers guests and relatives, e.g., by jumping on them, nipping them |  |
| 20 | F-H | I am concerned to release it in the presence of a toddler (I'm afraid it will knock the toddler over) |  |
| 21 | F-H | It disrupts mobility in the home/garden |  |
|  |  | In the case of your dog, to what extent are the following problems (if they occur) attributable to the dog’s aggression? (For example, in the case of the first question, to what extent is your dog's aggression responsible for your concern about releasing it?) |  |
| 22 | F-A | Cannot be released in an unfamiliar place | (0) There is no such problem –  (0) It is a problem, but not a result of aggression – (1) It is a problem, and, to some extent, is a result of aggression – (2) It is a problem, and, more or less, is a result of aggression – (3) It is a problem, and is largely a result of aggression |
| 23 | F-A | Is unable to engage in activities with other dogs in a conflict-free manner, e.g., play with them, get to know them |  |
| 24 | F-A | It learns with difficulty and slowly, as indicated, for example, by it lagging behind in dog school |  |
| 25 | F-A | Other dog owners don't like walking with us |  |
| 26 | F-A | Annoys, bothers guests and relatives, e.g., by jumping on them, nipping them |  |
| 27 | F-A | I am concerned to release it in the presence of a toddler (I'm afraid it will knock the toddler over) |  |
| 28 | F-A | It disrupts mobility in the home/garden |  |
|  |  | How typical are the following statements of your dog? |  |
| 29 | F-V | Cannot be quiet, whines or barks a lot even when there is nothing special to evoke this. | (0) Never – (1) Rarely – (2) Often – (3) Very often |
| 30 | F-V | If your dog starts to bark or whine, it is difficult to silence him/her. |  |

**Appendix E**

The trainer-report form of the Dog ADHD and Functionality Rating Scale (DAFRS; Aim 5). Space was provided for alternative answers where necessary. Asterisk-marked questions are not mandatory to be answered. Q: questions, R: reverse-worded and reverse-scored items; IA = inattention; H = hyperactivity; I = Impulsivity. Functionality item categorisation by the related impairments: F-IA = functionality-inattention; F-H: functionality-hyperactivity; F-I = functionality-impulsivity; F-A = functionality-aggression; F-V: functionality-vocalization. R = Reverse scored items.

| Description of the questionnaire for trainers | | | |  |
| --- | --- | --- | --- | --- |
| Dear Dog Trainer! The main goal of our research is to develop a new questionnaire to measure dogs' level of attention, activity, and impulsivity. Completing the questionnaire takes approximately 15 minutes, and we hope you will find it interesting! There are no right or wrong answers, but please, answer all questions to the best of your knowledge! Our goal is to get the most accurate picture of the dog. Please complete the questionnaire for one dog at a time. If you want to complete it for additional dogs, please re-open the questionnaire and submit a new response. We need a trainer's opinion since they have a broader perspective and more experience in dog behaviour, so they can better detangle extremely active, impulsive, inattentive dogs from dynamic, passionate or poorly motivated dogs. It is very important that the dog's owner also have to fill out the questionnaire, but please do not discuss the answers with each other. Data are stored for the sole purpose of communication regarding participation in research and are not communicated to any third parties. Thank you for helping us! | | | |  |
| Consent statement and privacy policy | | | |  |
| After data are processed and analysed, results will be published in the form of scientific presentations, papers, or educational materials. During data processing, data are stored at the Department of Ethology at Eötvös Loránd University. Research participants are informed about results upon reasonable request. Participation in this research is voluntary and anonymous, and the data obtained is used for scientific purposes only. In compliance with relevant data protection laws, personally identifying data are treated confidentially and stored separately from the rest of research data. Data sets shared with other researchers or made publicly available do not contain identifying information. Research data are further handled as part of a large database. Should you have any questions or comments regarding this research, please, contact our research team: Barbara Csibra, PhD student, ELTE Department of Ethology, email: csibrabarbara@gmail.com | | | |  |
| Consent Statement: I have read the consent and privacy policy information and am aware of the goals of- and processes involved in the current research. By participating in this research, I consent to my anonymized data being shared and treated as part of a larger database. I am aware that I may discontinue participating in this research at any time. | | | Yes/No (The questionnaire is terminated if you answer "No.") |  |
| GENERAL QUESTIONS | | | |  |
| Q/R | Subscale/Facet | Item description | Scale and scoring |  |
| 1 |  | Trainer's name (or unique identifier) (Data are stored for the sole purpose of communication regarding participation in research and are not communicated to any third parties.) |  |  |
| 2 | - | Owner's name (or unique identifier) (Data are stored for the sole purpose of communication regarding participation in research and are not communicated to any third parties.) | Text |  |
| 3 | - | Email address (It is used only for the communication necessary for the tests and is not passed on to third parties.) | Text |  |
| 4 | - | The trained dog's name | Text |  |
| 5 | - | The trained dog's sex | Male - female |  |
| 6 | - | How long have you been training the dog? | 1-2 months – 2-6 months – 6-12 months – more than 1 year |  |
| 7 | - | When was the last time you trained the dog? | I am still training the dog – 1-2 months ago – 2-6 months ago – 6-12 months ago – more than 1 year ago |  |
| QUESTIONS ON ATTENTION, ACTIVITY, IMPULSIVITY | | | |  |
|  |  | How typical are the following statements of the dog? |  |  |
| 1 | H | Fidgets, bustles. | (0) Never – (1) Rarely – (2) Often – (3) Very often – I don’t know (-) |  |
| 3 | I | Becomes very excited when facing a new, mildly stressful situation (e.g., facing new situation/place or meeting new people/dogs). |  |  |
| 4 | I | Once the dog "gets going", it is difficult to hold him/her back or stop. |  |  |
| 7 | I | Has no self-control. |  |  |
| 12 | IA | Has difficulties concentrating. |  |  |
| 13 | I | Is excessive, unrestrained, rampant. |  |  |
| 14 | H | Is rarely calm, even in familiar places, calm situations (e.g., at home). |  |  |
| 17 | I | Is difficult to calm down. |  |  |
| 20 | H | Active even after fatiguing or hard exercise/ work. |  |  |
| 24 | IA | Has difficulties with learning, because he/she does not pay attention. |  |  |
| 25 | IA | When the dog is asked to perform a task, he/she is reluctant to comply or withdraws from the situation. |  |  |
| 26 | IA | Performs poorly on tasks that require a lot of thinking. |  |  |
| 27 | IA | Has difficulties (makes many mistakes) with completing complicated tasks, even if he/she is familiar with the task and has frequently practiced he/she before. |  |  |
| 29 | IA | Quickly loses intertest. |  |  |
| 33 | H | Would always play and run. |  |  |
| 38 | I | Is difficult to control and handle. |  |  |
| 42 | I | Reacts rashly to new stimuli, without considering the consequences. |  |  |
| QUESTIONS ON FUNCTIONALITY | | | | |
|  |  | In the case of the dog, to what extent are the following problems (if they occur) attributable to the dog’s inattention? (For example, in the case of the first question, to what extent is the dog's inattention responsible for your concern about releasing it?) |  | |
| 1 | F-IA | Cannot be released in an unfamiliar place | (0) There is no such problem –  (0) It is a problem, but not a result of inattention – (1) It is a problem, and, to some extent, is a result of inattention – (2) It is a problem, and, more or less, is a result of inattention – (3) It is a problem, and is largely a result of inattention | |
| 2 | F-IA | Is unable to engage in activities with other dogs in a conflict-free manner, e.g., play with them, get to know them |  |  |
| 3 | F-IA | It learns with difficulty and slowly, as indicated, for example, by it lagging behind in dog school |  |  |
| 4 | F-IA | Other dog owners don't like walking with us |  |  |
| 5 | F-IA | Annoys, bothers guests and relatives, e.g., by jumping on them, nipping them |  |  |
| 6 | F-IA | I am concerned to release it in the presence of a toddler (I'm afraid it will knock the toddler over) |  |  |
| 7 | F-IA | It disrupts mobility in the home/garden |  |  |
|  |  | In the case of the dog, to what extent are the following problems (if they occur) attributable to the dog’s impulsivity? (For example, in the case of the first question, to what extent is the dog's impulsivity responsible for your concern about releasing it?) |  | |
| 8 | F-I | Cannot be released in an unfamiliar place | (0) There is no such problem –  (0) It is a problem, but not a result of impulsivity – (1) It is a problem, and, to some extent, is a result of impulsivity – (2) It is a problem, and, more or less, is a result of impulsivity – (3) It is a problem, and is largely a result of impulsivity | |
| 9 | F-I | Is unable to engage in activities with other dogs in a conflict-free manner, e.g., play with them, get to know them |  |  |
| 10 | F-I | It learns with difficulty and slowly, as indicated, for example, by it lagging behind in dog school |  |  |
| 11 | F-I | Other dog owners don't like walking with us |  |  |
| 12 | F-I | Annoys, bothers guests and relatives, e.g., by jumping on them, nipping them |  |  |
| 13 | F-I | I am concerned to release it in the presence of a toddler (I'm afraid it will knock the toddler over) |  |  |
| 14 | F-I | It disrupts mobility in the home/garden |  |  |
|  |  | In the case of the dog, to what extent are the following problems (if they occur) attributable to the dog’s excessive activity? (For example, in the case of the first question, to what extent is the dog's excessive activity responsible for your concern about releasing it?) |  | |
| 15 | F-H | Cannot be released in an unfamiliar place | (0) There is no such problem –  (0) It is a problem, but not a result of excessive activity – (1) It is a problem, and, to some extent, is a result of excessive activity – (2) It is a problem, and, more or less, is a result of excessive activity – (3) It is a problem, and is largely a result of excessive activity | |
| 16 | F-H | Is unable to engage in activities with other dogs in a conflict-free manner, e.g., play with them, get to know them |  |  |
| 17 | F-H | It learns with difficulty and slowly, as indicated, for example, by it lagging behind in dog school |  |  |
| 18 | F-H | Other dog owners don't like walking with us |  |  |
| 19 | F-H | Annoys, bothers guests and relatives, e.g., by jumping on them, nipping them |  |  |
| 20 | F-H | I am concerned to release it in the presence of a toddler (I'm afraid it will knock the toddler over) |  |  |
| 21 | F-H | It disrupts mobility in the home/garden |  |  |
|  |  | In the case of the dog, to what extent are the following problems (if they occur) attributable to the dog’s aggression? (For example, in the case of the first question, to what extent is the dog's aggression responsible for your concern about releasing it?) |  | |
| 22 | F-A | Cannot be released in an unfamiliar place | (0) There is no such problem –  (0) It is a problem, but not a result of aggression – (1) It is a problem, and, to some extent, is a result of aggression – (2) It is a problem, and, more or less, is a result of aggression – (3) It is a problem, and is largely a result of aggression | |
| 23 | F-A | Is unable to engage in activities with other dogs in a conflict-free manner, e.g., play with them, get to know them |  |  |
| 24 | F-A | It learns with difficulty and slowly, as indicated, for example, by it lagging behind in dog school |  |  |
| 25 | F-A | Other dog owners don't like walking with us |  |  |
| 26 | F-A | Annoys, bothers guests and relatives, e.g., by jumping on them, nipping them |  |  |
| 27 | F-A | I am concerned to release it in the presence of a toddler (I'm afraid it will knock the toddler over) |  |  |
| 28 | F-A | It disrupts mobility in the home/garden |  |  |
|  |  | How typical are the following statements of the dog? |  | |
| 29 | F-V | Cannot be quiet, whines or barks a lot even when there is nothing special to evoke this. | (0) Never – (1) Rarely – (2) Often – (3) Very often | |
| 30 | F-V | If your dog starts to bark or whine, it is difficult to silence him/her. |  |  |

**Figure S1.** Mean item rating distributions for the three ADHD factors (Aim 3).

**
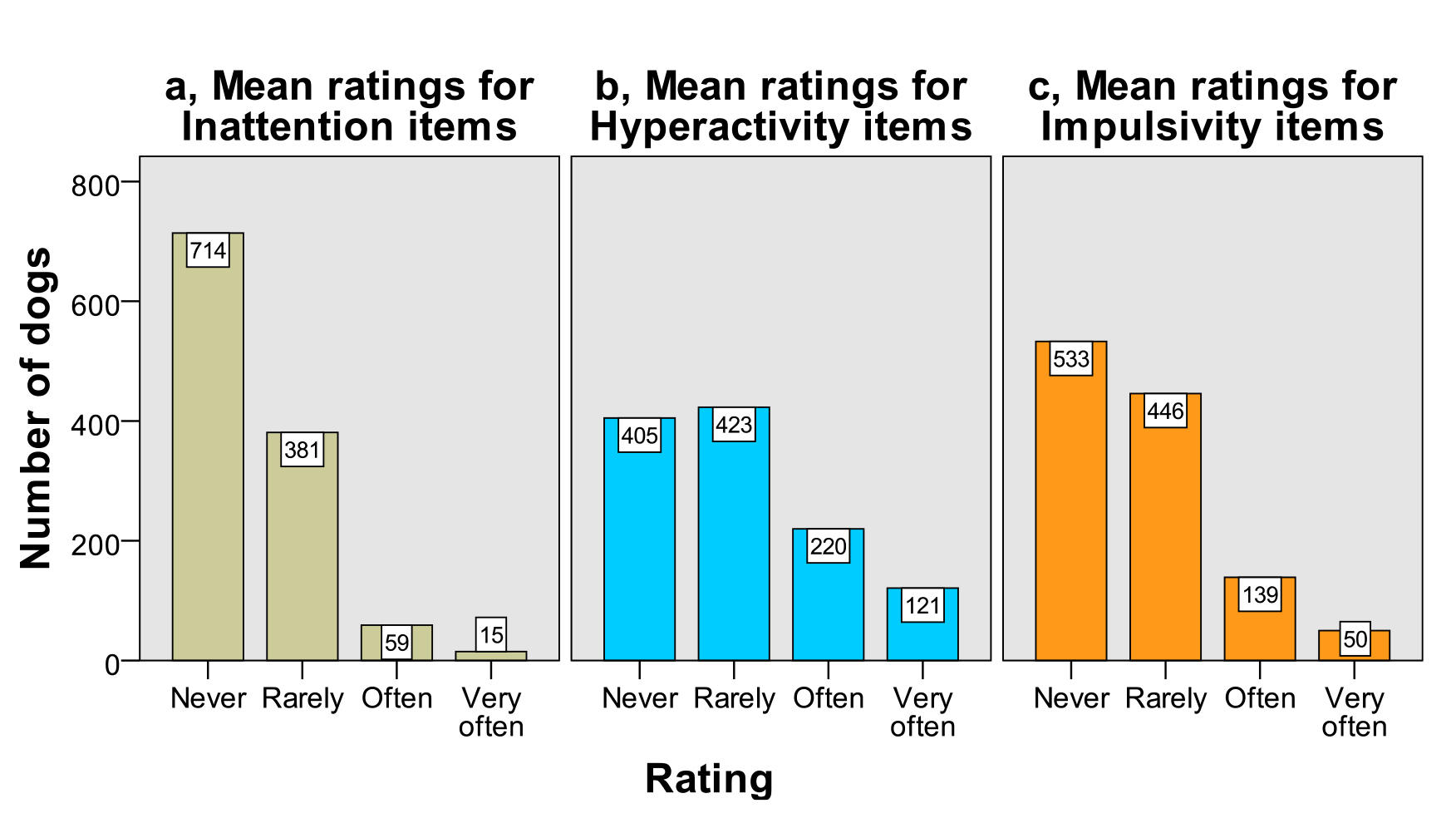
**

**Figure S2.** Owner and trainer rating (N=70) distributions of subscale total scores for inattention (a), hyperactivity (b), impulsivity (c) and distribution of ADHD total scores (d) (Aim 5).


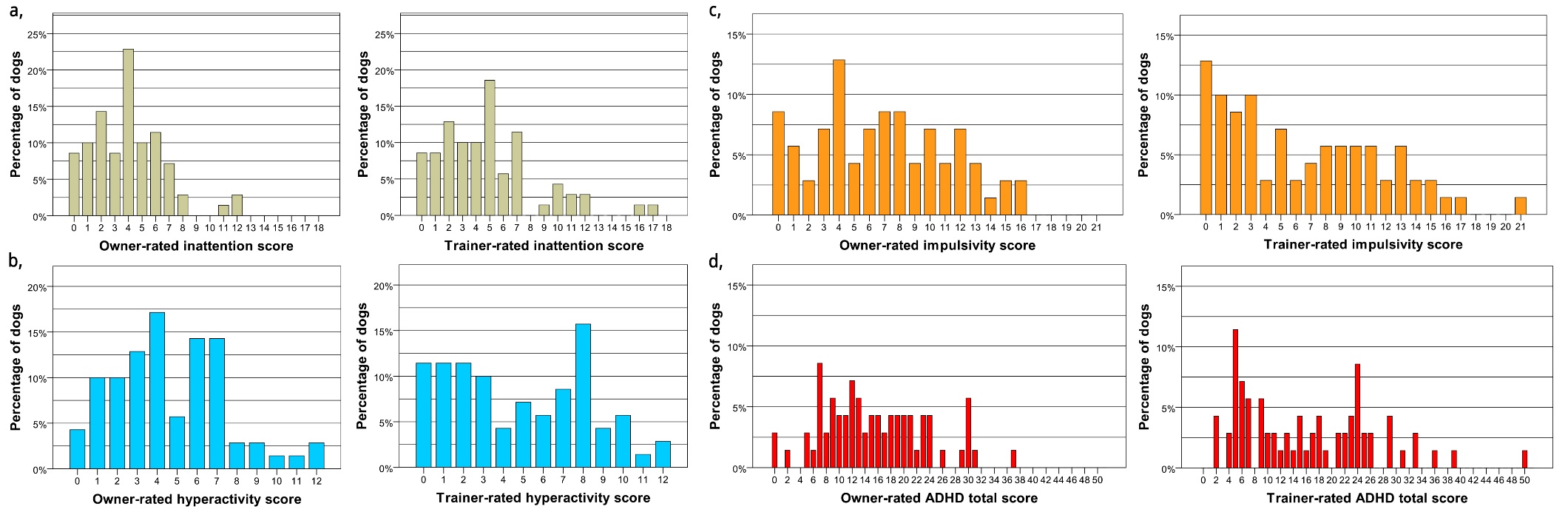


Note. The range of subscale scores after the final factor structure: inattention: 0-18 points, hyperactivity: 0-12 points, impulsivity: 0-21 points, ADHD total score:0-51 points.
